# Unlocking the Oxidative Performance of Peracetic Acid: A Comprehensive Review of Activation Pathways and Mechanisms for Environmental Remediation

**DOI:** 10.3390/toxics14010006

**Published:** 2025-12-19

**Authors:** Chun Xiao, Lihong Ai, Jinxi Chen, Wu Ren, Jinran Feng, Yue Lu, Yaoyao Chen, Yunxiu Luo, Xindong Yang, Min Dai, Jiangfei Cao, Jianqiao Qin, Chunsheng Xie

**Affiliations:** 1 College of Environmental and Chemical Engineering, Zhaoqing University, Zhaoqing 526061, China; 13554301718@163.com (C.X.);; 2 Guangdong Provincial Key Laboratory of Eco-Environmental Studies and Low-Carbon Agriculture in Peri-Urban Arease, Zhaoqing University, Zhaoqing 526061, China; 3 New Energy and New Materials Research Center, Zhaoqing University, Zhaoqing 526061, China; 4 College of Mechanical and Automotive Engineering, Zhaoqing University, Zhaoqing 526061, China

**Keywords:** peracetic acid, activation, active radicals, environmental remediation, by-products

## Abstract

The activation of peracetic acid (PAA) to generate highly reactive species has emerged as a promising advanced oxidation process (AOP) for the degradation of refractory organic pollutants. This review systematically summarizes the recent advancements in PAA-based AOPs, encompassing various activation strategies, underlying reaction mechanisms, and applications across different environmental matrices. The activation methods are critically discussed, including direct energy activation, homogeneous catalysis, and heterogeneous catalysis. The generation process of diverse reactive species, like hydroxyl radicals (HO·), organic radicals (CH_3_C(O)O·, CH_3_C(O)OO·), and singlet oxygen (^1^O_2_), was introduced, and their oxidation selectivity and anti-interference ability were compared. Furthermore, the practical applications of PAA-based AOPs in treating wastewater, groundwater, and contaminated soil/sediments are reviewed. Finally, this review outlines critical challenges, including potential toxic byproduct formation, catalyst stability, and economic feasibility, and proposes future research directions to facilitate the transition of PAA-based AOPs from laboratory-scale research to full-scale implementation. This review provides insights for developing efficient, selective, and sustainable oxidation technologies, thereby contributing to the mitigation of emerging contaminant threats and the advancement of environmental remediation practices.

## 1. Introduction

In recent decades, the increasing discharge of agricultural non-point source pollutants, industrial manufacturing wastewater, and urban sewage has exacerbated water scarcity and pollution, making it a hotspot of global concern. Recently, the focus of research into water pollutants has moved from traditional substances such as heavy metal ions and persistent organic pollutants to emerging contaminants (ECs) [1]. Concurrently, advancements in technology and detection capabilities are expected to classify more pollutants as ECs [2]. These substances pose significant threats to human health due to their chemical stability, resistance to biodegradation, and potential for bioaccumulation [3]. Furthermore, ECs can be detected even at trace levels with improved analytical sensitivity [4,5,6]. However, conventional water treatment processes exhibit limited removal capacity for ECs, leading to challenges such as incomplete degradation, secondary pollution, and high economic costs. Therefore, there is an immediate requirement for the development of advanced treatment technologies for ECs to achieve sustainable development more effectively.

Advanced oxidation processes (AOPs) exhibit high efficiency for the treatment of wastewater, and AOPs have been proven effective for degrading ECs through the generation of reactive oxygen species (ROS) [7,8,9]. The degradation mechanism of most AOPs is to generate hydroxyl radicals (HO·), sulfate radicals (SO_4_^−^), superoxide anion radicals (O_2_^−^), and singlet oxygen (^1^O_2_) via different activation methods from hydrogen peroxide (H_2_O_2_), peroxydisulfate (PDS) and peroxymonosulfate (PMS). The main sources of ROS are the homolytic cleavage of the peroxy bond (O-O) [10]. Among these, HO· exhibit high redox potential (E_0_ = 1.9~2.8 V), which can remove the target pollutants non-selectively [11,12]. However, the non-selectivity of HO· makes it susceptible to reaction with water matrix components (carbonate, bicarbonate, and natural organic matter (NOM)), decreasing the degradation efficiency for target pollutants in complex water matrices [7]. Consequently, reactive species with higher selectivity than HO· are gradually gaining attention [13,14].

**Figure 1:**
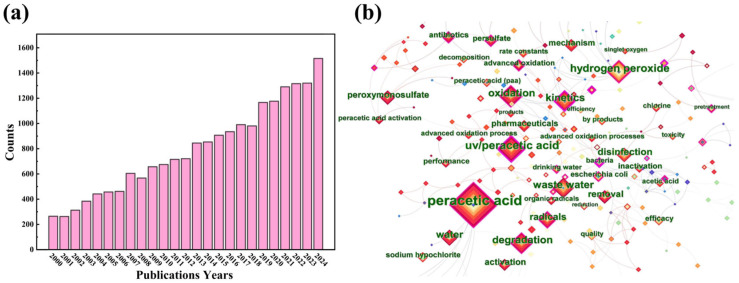
(**a**) Number of PAA-related publications in Web of Science from 2000 to 2022; (**b**) collinearity analysis of PAA keywords reported in the past decade, with keywords including ‘peracetic acid’, ‘peroxyacetic acid’, or ‘peroxyacetic acid’.

In the last few years, peracetic acid (PAA)-based AOPs have attracted keen attention due to the high efficiency and environmental friendliness. PAA-based AOPs generate HO·, methyl radicals (CH_3_·), acetoxyl radicals (CH_3_C(O)O·), acetylperoxyl radicals (CH_3_C(O)OO·), and other active species. They exhibit good anti-interference capability and minimal byproduct formation, making PAA an excellent oxidant for water pollution treatment [15,16,17,18,19]. As shown in Figure 1a, the research about PAA activation has become a hot topic, with a significant increase in publications indexed in “Sci-Expanded” of the “Web of Science” database since year 2000. Retrieved in the core collection of Web of Science, specific search query conditions include (TS = (“peracetic acid” OR “peroxyacetic acid”)), the range of literature publication year (2000–2022), and the use of CiteSpace for keyword co-presentation analysis. The results of co-occurrence analysis indicated that disinfection, organic pollutant removal, wastewater treatment, and advanced oxidation are major research directions in the past decades (Figure 1b). Additionally, PAA-based AOPs involved combinations with homogeneous technologies like persulfate, H_2_O_2_, sodium hypochlorite, and UV, and the discussions of organic radicals and synergistic effects. The rapid emergence of PAA activation as a superior AOP can be seen, yet the field lacks a comprehensive and critical synthesis that connects fundamental reaction mechanisms across diverse activation methods with their practical applications in various environmental matrices.

**Figure 2:**
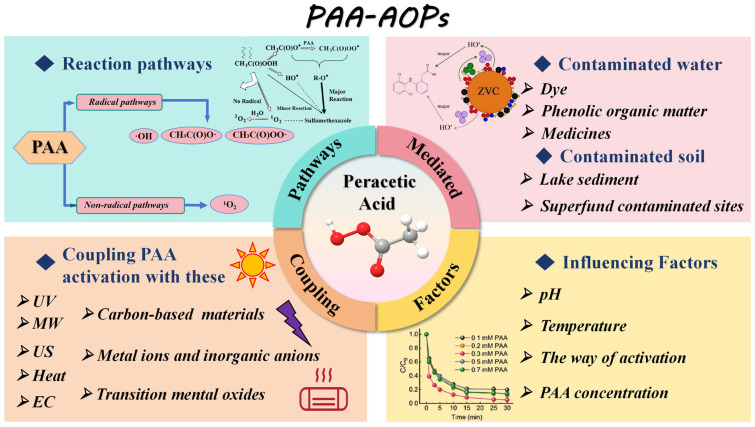
Systemic overview diagram of PAA-based AOPs.

Herein, as shown in Figure 2, this review systematically summarizes activation methods of PAA, including direct activation (microwave, ultraviolet, ultrasound, heat, and electrochemistry), homogeneous catalytic activation (metal ions and inorganic anions), and heterogeneous catalytic activation (metal catalysts and non-metallic catalysts). In addition, the degradation mechanisms of PAA-based AOPs for the treatment of organic pollutants were introduced. Next, the application status and progress of PAA-based AOPs in wastewater, soil, groundwater, and disinfection fields were summarized. More interestingly, the operational feasibility and economic cost of PAA activation were analyzed. Finally, the existing problems of PAA-based AOPs and the direction that needs to be focused on in the future were pointed out.

## 2. Properties of PAA

PAA is a colorless liquid with a pungent odor similar to that of acetic acid. It is typically prepared by reacting acetic acid (CH_3_COOH) and H_2_O_2_ under sulfuric acid catalysis (Equation (1)) [20,21]. As depicted in Figure S1, the structure of PAA is composed of C, H, and O, which is characterized by an O-O bond similar to H_2_O_2_. The value of the bond dissociation energy of the PAA peroxy bond is 170 kJ/mol, which is slightly weaker than that of H_2_O_2_ (213 kJ/mol). It shows that PAA easily cleavages the O-O band to produce a more highly reactive species than H_2_O_2_. Past studies have shown that the stability of PAA is closely related to solution pH, decomposing readily under alkaline conditions. The spontaneous decomposition of PAA occurs with low hydrolysis within the pH range of 5.5–8.2 (Equation (2)) [21]. Conversely, PAA undergoes simultaneous decomposition and hydrolysis at pH values greater than 8.2 (Equation (3)), resulting in the production of acetic acid and H_2_O_2_ [22,23]. PAA is relatively unstable, often stored at 4 °C in thick polyethylene bottles.

CH_3_COOH + H_2_O_2_ → CH_3_C(O)OOH + H_2_O (1)

2CH_3_C(O)OOH → CH_3_COOH + H_2_O (2)

CH_3_C(O)OOH + H_2_O → CH_3_COOH + H_2_O_2_ (3)

Table 1 summarizes the physicochemical properties of main oxidants in AOP, including PAA, H_2_O_2_, PMS, and PDS [11,14,24,25,26]. PAA exhibits low boiling and melting points, high solubility in various solvents, and significant volatility and instability, necessitating careful storage and handling. The redox potential range of PAA is 1.06~1.96 V, increasing with the solution pH. In comparison, H_2_O_2_ shows a higher density and superior solubility in polar organic solvents, while PMS and PDS possess greater molar masses and thermal stability, though with more limited solubility, being confined mainly to aqueous systems. These distinct properties critically inform the selection of an appropriate oxidant for specific AOP applications.

## 3. PAA Activation Technologies and Mechanisms

### 3.1. Direct Activation

#### 3.1.1. Microwave (MW) Activation

**Figure 3:**
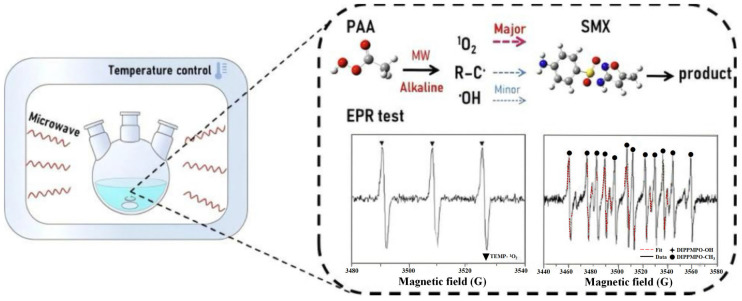
Degradation mechanism of SMX in MW/PAA system [27].

The traditional thermal activation method via convective heat transfer suffers from slow startup and uneven heating. Microwave heating is based on the interaction between the electric field and molecular dipoles, thereby ensuring more uniform heating. DAI et al. found that there was a significant non-free radical path in the PAA activation system through EPR and quenching experiments [27]. As shown in (Equations (4)–(8)), this pathway was characterized by the predominant generation of ^1^O_2_, which subsequently led to the production of R-O· through radical reaction. As shown in Figure 3, these active species can effectively degrade sulfamethoxazole SMX. The system responds quickly, but energy efficiency depends on the ability of the reaction system to absorb microwaves.

(4) $${\mathrm{CH}}_{3}C(O)\mathrm{OH}\;\overset{MW}{\to }\;{\mathrm{CH}}_{3}C(O)O\cdot +\cdot \mathrm{OH}$$

CH_3_C(O)O· → ·CH_3_ + CO_2_ (5)

CH_3_C(O)O· + CH_3_C(O)OOH → CH_3_C(O)OO· + CH_3_C(O)OH (6)

·OH + CH_3_C(O)OOH → CH_3_C(O)OO· + H_2_O (7)

(8) $${\mathrm{CH}}_{3}C(O)\mathrm{OOH}+{\mathrm{CH}}_{3}C(O){\mathrm{OO}}^{-}\;\overset{MW}{\to }\;{\mathrm{CH}}_{3}C(O)\mathrm{OH}+{\mathrm{CH}}_{3}C(O)O^{-}$$

#### 3.1.2. Ultraviolet Light (UV) Activation

**Figure 4:**
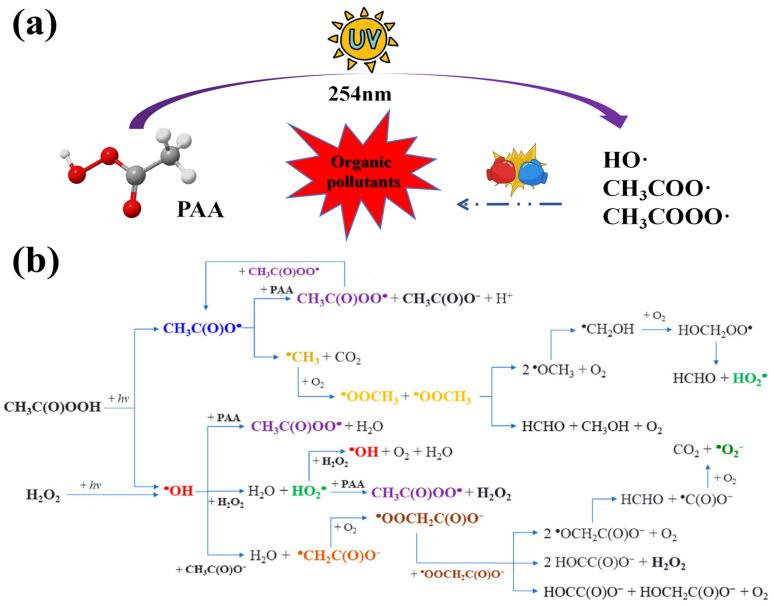
(**a**) UV/PAA reaction system; (**b**) UV/peracetic acid reaction pathway.

The UV activation method is notable for its simplicity, operational ease, and scalability. As depicted in Figure 4a, many oxidative species were generated to inactivate bacteria and degrade organic pollutants in UV/PAA systems [28]. Firstly, UV homolytically cleaved the O-O bond of PAA, producing CH_3_C(O)O· and HO·. Next, CH_3_C(O)O· rapidly decarboxylated to ·CH_3_ and CO_2_; ·CH_3_ reacted with dissolved oxygen and formed CH_3_O_2_·. In addition, HO· also reacted with PAA, generating CH_3_C(O)O·, which decomposes to ·CH_3_. CH_3_C(O)O· can also abstract H from PAA, forming CH_3_C(O)OO· and acetic acid. Liu Wen et al. [29] established an in situ EPR detection method combined with DFT calculation, which accurately identified the free radicals generated in the UV/PAA activation system, and clarified their free radical formation mechanism. The degradation reactions are shown in (Equations (9)–(12)) [24,30]. Furthermore, the detailed degradation process is displayed in Figure 4b.

CH_3_C(O)OOH + hv → CH_3_C(O)O· + HO· (9)

H_2_O_2_ + hv → 2HO· (10)

CH_3_C(O)OOH + CH_3_C(O)O· → CH_3_C(O)OO· + CH_3_C(O)OH (11)

CH_3_C(O)OOH + HO· → CHC(O)OO· + H_2_O (12)

CAI et al. [31] showed that the UV/PAA system was effective at degrading antibiotics under 254 nm UV irradiation. Building on this, YAN et al. [32] found that the HO· generated by the UV/PAA system enhanced the removal and mineralization of oxytetracycline under 254 nm wavelength and 223.2 mJ/cm^2^ irradiation dose. It also found that the degradation rate of the UV/PAA system was twice that of UV and PAA alone. Scholars have also studied the key influencing factors of the UV/PAA system. The results revealed that UV wavelength significantly affects the yield of active substances. The shorter wavelengths with higher energy levels favored more active substance formation. Additionally, the reaction rate constantly showed an increase when UV irradiation intensity increased. Similarly, the PAA dosage has an optimal value in the UV/PAA system. An insufficient PAA dosage results in an inadequate yield of reactive species and consequently weak oxidative degradation efficiency, whereas an excess of PAA may scavenge radicals, thereby diminishing pollutant removal efficiency [33]. Nevertheless, the dependence on artificial illumination remains a key limitation, leading to the significant operating cost of wastewater treatment that hinders the large-scale implementation of the UV/PAA system.

#### 3.1.3. Ultrasound (US) Activation

**Figure 5:**
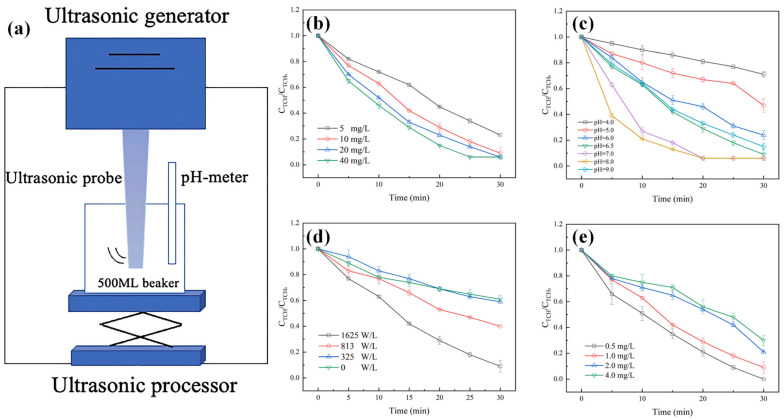
(**a**) Schematic diagram of US/PAA system, (**b**) the effect of PAA dosage, (**c**) ultrasonic power, (**d**) pH, and (**e**) initial TCH concentration on TCH degradation efficiency in US/PAA system [34].

US technology is a green and safe water treatment technology with broad application prospects. As shown in Figure 5a, the formation and growth of cavities followed by their violent collapse generate high temperatures within and around the cavities in the US/PAA system [34]. Meanwhile, the energy released during the collapse activates free radicals. Previous studies on US activation for target pollutant degradation have been extensively documented [35,36]. Radical quenching experiments and electron paramagnetic resonance (EPR) proved that ^1^O_2_ and organic RO^−^ radicals were the primary active species in US/PAA processes, as illustrated in (Equations (13)–(15)).

CH_3_C(O)OOH + US → CH_3_C(O)OO· + H· (13)

CH_3_C(O)OOH + CH_3_C(O)OO· → CH_3_C(O)O· + CH_3_C(O)OH + ^1^O_2_ (14)

CH_3_C(O)OOH + US → CH_3_C(O)O· + HO· (15)

Yao et al. [34] established the US/PAA system to investigate the removal efficiency of total hydrocarbons (TCHs) in wastewater. The experimental results revealed that the degradation efficiency of TCH reached 99.4% within 30 min, achieving a reaction rate of 4.88 times that of conventional PAA processes. In addition, they also systematically studied the influencing factors in the process of US/PAA degradation. As shown in Figure 5b, the degradation efficiency of TCH exhibited a positive correlation with the PAA dosage, increasing from 77.0% to 99.4% as the concentration was raised from 5 to 40 mg/L. The degradation efficiency was also found to be highly dependent on solution pH (Figure 5c). Under acidic to neutral conditions (pH < 8.2), PAA demonstrated greater oxidative potency and stability, leading to superior TCH degradation. Furthermore, the ultrasonic energy input played a critical role, elevating the power density from 325 to 1625 W/L intensified cavitation, thereby promoting the generation of free radicals and increasing TCH removal efficiency from 41.0% to 99.4% within 30 min (Figure 5d). Conversely, the degradation efficiency gradually declined from 99.9% to 77.4% with an increase in the initial TCH concentration (Figure 5e), indicating a potential limitation related to the availability of the US/PAA system at higher pollutant concentrations.

#### 3.1.4. Thermal Activation

**Figure 6:**
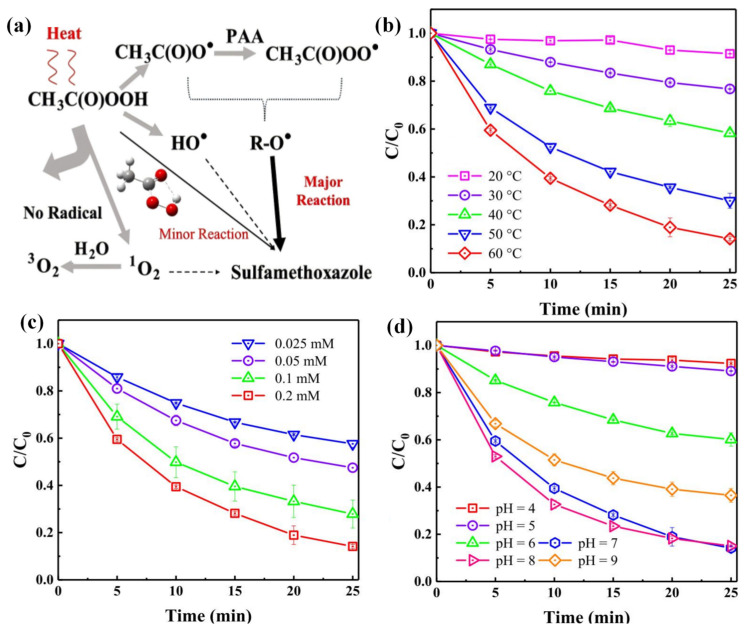
(**a**) Degradation mechanism of heat/PAA system; the effect of temperature; (**b**) PAA dosage; (**c**) pH; (**d**) the degradation of SMX via thermally activated PAA [37].

PAA contains weak O-O bonds whose cleavage can be accelerated by heat. As shown in Figure 6a, the thermal activation of PAA involved a radical pathway and non-radical pathway. These pathways generate reactive species such as ^1^O_2_, HO· and R-O· [37,38]. The radical pathway primarily involved the thermal activation of PAA to generate HO· and CH_3_C(O)O·, as illustrated in (Equation (16)). The non-radical pathway involved the cleavage of PAA induced by thermal energy to produce ^1^O_2_, as illustrated in (Equation (17)).

(16) $${\mathrm{CH}}_{3}C(O)\mathrm{OOH}\;\overset{H\mathrm{eat},\;60{}^{\circ}C}{\to }\;{\mathrm{CH}}_{3}C(O)O\cdot +\mathrm{HO}\cdot$$

(17) CH3C(O)OOH+CH3C(O)OO− →Heat, 60 °C CH3C(O)OH+CH3C(O)O−+O21

Wang et al. [37] systematically evaluated the degradation of SMX via thermally activated PAA. As illustrated in Figure 6b, the reaction temperature from 20 to 60 °C markedly enhanced SMX removal, achieving 86% degradation efficiency within 25 min at 60 °C. In addition, the degradation efficiency decreased with increasing PAA concentration (0.025–0.2 mM) (Figure 6c), suggesting that excess oxidants can scavenge potential free radicals. Furthermore, the degradation process exhibited strong pH dependence (Figure 6d). The highest efficiency occurred at neutral to slightly basic conditions (pH 7–8), with removal efficiencies of 86% and 85%. In contrast, acidic conditions (pH 4–5) severely inhibited the degradation reaction, highlighting the critical role of pH in mediating reactive species formation and stability.

#### 3.1.5. Electrochemical Activation

**Figure 7:**
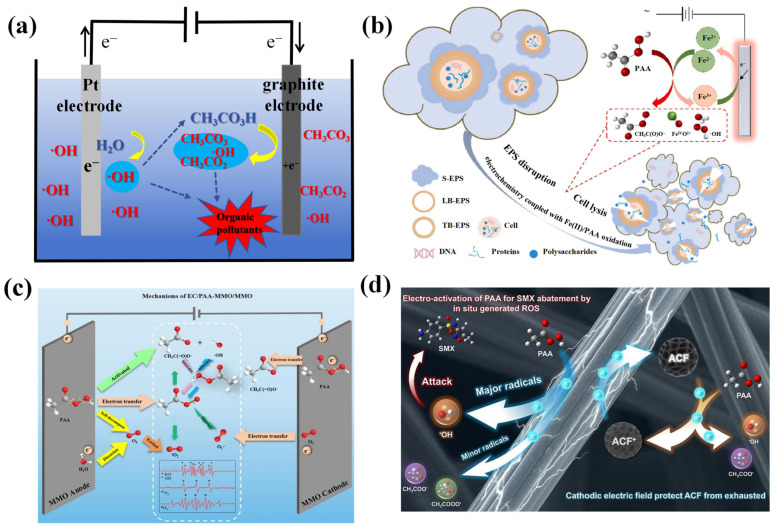
(**a**) The degradation mechanism of MB in the EC/PAA system; (**b**) the decomposition mechanism of waste-activated sludge in the EC/Fe(II)/PAA system [42]; (**c**) the degradation mechanism of PPL in the EC/PAA-MMO/MMO system [43]; (**d**) the degradation mechanism of SMX in the E-ACF-PAA system [44].

Electrochemical oxidation technology demonstrates promising applications in water treatment and disinfection due to its strong oxidizing capacity and reduced secondary pollution characteristics [39]. As illustrated in Figure 7a [22], combined electrochemical oxidation (EC) with PAA was used to construct the EC/PAA synergistic system. The anode generated HO· and R-O· radicals, while the cathode provided electrons to activate PAA, which enhanced mass transfer efficiency and prolonged the redox reaction duration for organic pollutant removal. This approach overcame the inherent limitations of EC and achieved efficient degradation of organic pollutants [40,41], as seen in (Equations (18)–(20)).

CH_3_C(O)OOH + e^−^ → CH_3_C(O)O· + OH^−^ (18)

CH_3_C(O)OOH + e^−^ → CH_3_C(O)O^−^ + HO· (19)

CH_3_C(O)OOH + HO· → CH_3_C(O)OO· + H_2_O (20)

As shown in Figure 7b, Zhang et al. [42] developed an EC/Fe(II)/PAA system to investigate the performance of decomposing waste-activated sludge. The results revealed that compared to the Fe(II)/PAA, the EC/Fe(II)/PAA system increased the levels of soluble chemical oxygen demand (COD), carbohydrates, and nucleic acids by 62.85%, 41.15%, and 12.21%, respectively. EC effectively accelerated the conversion of Fe(III) to Fe(II), which facilitated PAA activation and significantly enhanced the resource utilization of sludge. As displayed in Figure 7c, Yang et al. [43] investigated the removal efficiency of propranolol (PPL) using a Ti/IrO_2_-Ta_2_O_5_ electrode as both the anode and the cathode. The experimental data demonstrated an 87.05% removal rate within 20 min. As seen in Figure 7d, PAN et al. [44] developed an electrochemical system using activated carbon fibers (E-ACF) as the cathode effectively activates PAA (E-ACF-PAA). Compared with conventional cathodes, the ACF demonstrated higher PAA activation efficiency and SMX removal efficiency. The applied cathode electric field could prevent PAA from oxidation, and the results of cycle experimental showed that the catalytic performance could be maintained for more than 50 cycles. Table 2 summarizes the technical characteristics of some direct PAA activation methods and compares their reaction mechanisms and degradation efficiency.

### 3.2. Homogeneous Catalytic Activation

Homogeneous catalytic activation is a key strategy to improve the oxidation ability of PAA. Transition metal ions rely on the redox cycle of metal species (such as Fe^2+^/Fe^3+^, Co^2+^/Co^3+^) to crack the O-O bonds in PAA and generate activity [46,47]. Oxygen species have become an important way to activate PAA to produce active species. In addition, inorganic anions can also activate PAA through nucleophilic attack or removal of free radicals, which will lead to the formation of secondary oxidants or free radical species [24,48].

#### 3.2.1. Metal Ions

**Figure 8:**
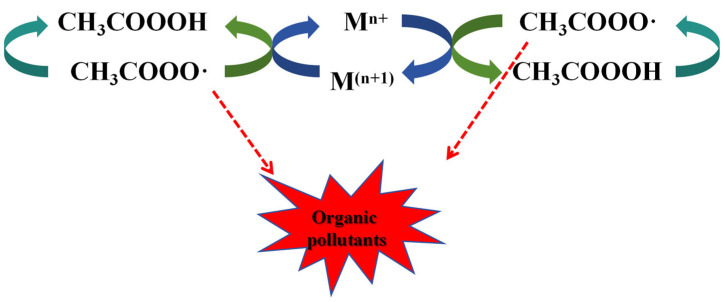
Mechanism degradation of transition metal ion/PAA system.

Transition metal ions are widely used in advanced oxidation processes due to natural abundance and low cost [49]. Many studies in the past have demonstrated the theoretical feasibility of the transition metal activation of PAA [50]. Cr [51], Fe [46], Co [47], Mn [52], and Ru [53] ions activated O-O cleavage to generate ROS for degrading pharmaceuticals (carbamazepine, diclofenac, and SMX), phenols (dichlorophenol and 4-chlorophenol), and dyes (methylene blue and acid orange). Homogeneous degradation reaction is simple and effective for various pollutants, but there are risks of metal leaching and secondary pollution [54]. Heterogeneous catalysts are easily recoverable and operate under mild conditions [55]. Analogous to the Fenton system (H_2_O_2_/Fe^2+^), the studies about PAA activation by transition metals (Fe, Co, Mn, Cu) have been reported. Wang et al. [56] found that transition metals could decompose the O-O bond and activate PAA to produce reactive species (Equations (21) and (22)); M stands for metal ion. In this mechanism, Mn^+^ and M^(n+1)+^ undergo internal cycling, aligning with the concept of green development (Figure 8) [54,57].

Mn^+^ + CH_3_C(O)OOH → M^(n+1)+^ + CH_3_C(O)O· + OH^−^ (21)

M^(n+1)+^ + CH_3_C(O)OOH → Mn^+^ + CH_3_C(O)OO· + H^+^ (22)

##### Cr^3+^ Ion

As illustrated in Figure S2a, Bell et al. [51] developed a Cr(III)/PAA system, and the reaction mechanism of the Cr(III)/PAA system was proposed. The key chemical processes of the system are showed in (Equations (23)–(26)). Furthermore, quenching experiments, probe molecule experiments, and EPR test confirmed that OH· radicals were the primary ROS, while Cr(IV)/Cr(V) served as auxiliary active components. Bell et al. [51] also used this system to investigate the degradation process of Trimethoprim (TMP). Experimental results demonstrated that under alkaline conditions, the Cr(III)/PAA system achieved a 90% degradation efficiency, significantly outperforming the individual PAA and H_2_O_2_/Cr(III) systems; the Cr(III)/PAA system’s TMP degradation process is illustrated in Figure S2b [51]. However, it is noteworthy that Cr(VI) is a highly toxic compound, which is widely recognized as both a human carcinogen and environmental pollutant, posing risks of secondary pollution to both organisms and ecosystems. Therefore, the application of the Cr(III)/PAA system in large-scale water treatment is limited.

CH_3_C(O)OH + Cr(III) → CH_3_C(O)O·+ Cr(VI) + OH^−^ (23)

CH_3_C(O)OH + Cr(III) → CH_3_C(O)O^−^ + Cr(VI) + ·OH (24)

CH_3_C(O)OH + Cr(VI) → CH_3_C(O)O^−^ + Cr(V) + ·OH (25)

CH_3_C(O)OOH + Cr(V) → CH_3_C(O)O· + Cr(VI) + OH^−^ (26)

##### Fe^3+^ Ion

J. Kim et al. [46] established an Fe(II)/PAA system to degrade MB, naproxen (NPX), and bisphenol A (BPA). The significant enhancement removal effect of the organic pollutant was observed compared to PAA alone. Reaction steps of the Fe(II)/PAA system are displayed in (Equations (27)–(29)). Additionally, Fe^2+^ can also react with H_2_O_2_ (Equations (30)–(32)), but the reaction rates were much lower than with PAA [46]. The results of degradation experiments demonstrated that the removal efficiencies of different pollutants reach 48% to 98% within pH 3.0–8.2 conditions. Wang et al. [58] created a novel heterogeneous Fe^2+^-modified zeolite/PAA system, which completely removed SMX within 50 min under neutral pH conditions. Virkutyte et al. [59] found that an eco-friendly magnetic iron oxide column-supported montmorillonite could activate PAA to generate radicals for the degradation of dichlorophenol (DCP), and the removal efficiency of DCP reached 70% within 3.5 h.

Fe^2+^ + CH_3_C(O)OOH → Fe^3+^ + CH_3_C(O)O·+ OH^−^ (27)

Fe^2+^ + CH_3_C(O)OOH → Fe^3+^+ CH_3_COO^−^ + ·OH (28)

Fe^2+^ + CH_3_C(O)OOH → FeIVO^2+^ + CH_3_C(O)OH (29)

Fe^2+^ + H_2_O_2_ → Fe^3+^ + ·OH + OH^−^ (30)

FeIVO^2+^ + H_2_O_2_ → FeIVO^2+^ + H_2_O (31)

Fe^3+^ + H_2_O_2_ → Fe^2+^+ ·HO_2_ + H^+^ (32)

##### Co^3+^ Ion

The first report was published on the activation of PAA by cobalt in year 1951 [60]. Co, as a transition metal ion, played a key role in the Co/PAA system for organic pollutants. Studies have demonstrated that Co exhibits superior catalytic performance in the activation of PAA compared to Mn, Fe, and Cu [61]. Notably, the main reactive species generated during the Co/PAA system were R-O·, with little HO· formation. Wang et al. [47] developed the Co/PAA system for SMX degradation. Experimental results indicated that Co ions could decompose PAA to generate acetyl peroxide radicals with strong oxidative capacity. After 15 min, the SMX removal efficiency reached 89.4%. Additionally, it was confirmed that high initial concentrations of furosemide, triclosan, and naproxen could all be effectively removed in the Co/PAA system. Kim et al. [57] evaluated the degradation efficiencies of carbamazepine (CBZ), SMX, NAP, and BPA in the Co/PAA system. Experimental results showed high degradation efficiency for four pollutants under the initial pH range of 3.0–8.1. The highest removal efficiencies of BPA, NAP, SMX, and CBZ were 100%, 100.0%, 98.5%, and 87.7%, respectively. The Co^2+^-activated PAA reaction steps were illustrated in (Equations (33) and (34)) [47].

Co^2+^ + CH_3_C(O)OOH → Co^3+^ + CH_3_C(O)O· + OH^−^ (33)

Co^3+^ + CH_3_C(O)OOH → Co^2+^ + CH_3_C(O)OO· + H^+^ (34)

The above results indicated that Co was recognized as an effective catalyst for the decomposition of PAA [62]. However, the Co-based homogeneous catalysts may pose potential health risks and cause secondary pollution to the environment, which limits practical applications. The current Co/PAA system faced challenges regarding the leaching of Co ions, toxicity risks, and poor recyclability. It suggested that, in the future, there should be a focus on developing composite Co-based catalysts and supported catalysts to enhance catalytic activity, stability, and recyclability.

##### Mn^2+^ Ion

Mn is a common metal catalyst, and past studies have explored the feasibility of Mn in AOPs [63]. It indicated that Mn^2+^ decomposed PAA via a non-radical pathway involving complex redox reaction steps [24]. Popov et al. [64] proposed non-radical reaction pathways as shown in (Equations (35)–(38)). Notably, the Mn-based complexes were observed to be the active species of the Mn/PAA system, not the free radical.

Mn^3+^ + 4CH_3_C(O)OOH + 2H_2_O → MnO_4_^−^ + 4CH_3_C(O)OH + O_2_ + 4H^+^ (35)

MnO_4_^−^ + Mn^2+^ → MnO_4_^2−^ + Mn^3+^ (36)

3MnO_4_^2−^ + 4H^+^ → MnO_2_ + 2MnO_4_^−^ + 2H_2_O (37)

Mn^2+^ + MnO_2_ + 4H^+^ → 2Mn^3+^ + 2H_2_O (38)

Rokhina et al. [30] investigated the phenol degradation efficiency of PAA in the presence of MnO_2_, revealing that peroxide bonds of PAA underwent complete dissociation, generating major reactive species such as HO· and R-O·. The experimental results demonstrated that the rate constant of the Mn^2+^/PAA system (k = 6.05 × 10^−2^ s^−1^) for degradation of Orange II was several orders of magnitude higher than the rate constant of the Mn^2+^/H_2_O_2_ system (k = 7.92 × 10^−4^ s^−1^) under the same reaction conditions. Additionally, the ultrasonic-assisted MnO_2_/PAA system for phenol degradation was reported, and it was found that both radical pathways and non-radical pathways may be involved [55].

##### Ru^3+^ Ion

Ru (III) has been utilized as a catalyst for PAA activation in organic synthesis, while Ru complexes have demonstrated catalytic capabilities for hydrogen and oxygen production [65,66]. In addition, Li et al. [67] developed a Ru(III)/PAA system to degrade micro-pollutants in wastewater. The activation mechanism of the Ru(III)/PAA system was elucidated and is detailed in (Equations (39)–(42)).

CH_3_C(O)OOH + Ru^3+^ → CH_3_C(O)O· + Ru^4+^ + OH^−^ (39)

CH_3_C(O)OOH + Ru^3+^ → CH_3_C(O)O- + Ru^4+^ + ·OH (40)

CH_3_C(O)OOH + CH_3_C(O)O· → CH_3_C(O)OO· + CH_3_COOH (41)

CH_3_C(O)OOH + ·OH → CH_3_C(O)OO· + H_2_O (42)

Li et al. [67] reported that the Ru(III)/PAA system achieved complete degradation of SMX within 2 min under neutral conditions in phosphate buffer (0.5–20.0 mM), significantly outperforming other metal activators such as Fe(II), Fe(III), Mn(II), Mn(III), Co(II), and Cu(II), which achieved only 20% removal efficiency of SMX under identical conditions. Furthermore, EPR and quenching studies identified acetyl peroxy radicals as the dominant reactive species responsible for SMX degradation. Importantly, the Ru(III)/PAA process exhibited strong resistance to common water matrix interferents, with negligible inhibition by chloride, carbonate, or phosphate ions, highlighting its robustness for potential applications in complex water chemistries.

The efficiency and reaction mechanisms of homogeneous transition metal ions, which were common catalysts in PAA systems, differ considerably. These characteristics are systematically summarized in Table 3, which serves as a valuable reference for selecting appropriate catalytic systems in future applications.

#### 3.2.2. Inorganic Anions

##### Cl^−^

The presence of common inorganic anions in aquatic matrices significantly influences the degradation efficiency of organic pollutants in PAA-based AOPs [24]. Given the typically high concentrations of Cl^−^ in wastewater, the impact proceeds through two primary pathways. Firstly, it directly reacts with PAA to form the secondary oxidant HOCl, as shown in (Equation (43)) [68]. Secondly, the primary radicals (HO· and R-O·) were scavenged to yield a suite of chlorinated radical species Cl^−^, ClOH, and Cl_2_, as detailed in (Equation (44)) [69,70]. Although these chlorine-centered radicals generally possess a lower redox potential compared to HO·, the reactivity is highly selective, leading to pollutant-specific effects.

Cl^−^ + CH_3_C(O)OOH → HOCl + CH_3_COO^−^ (43)

Cl^−^ + ·OH → HOCl·^−^ (44)

Consequently, the influence of Cl^−^ was strongly dependent on the target contaminant’s molecular structure [24]. Chen et al. [69] reported negligible inhibition by Cl^−^ (up to 200 mM) on naproxen degradation in a UV/PAA system, while Zhang et al. [70] observed minimal effect on diclofenac (DCF) removal at Cl^−^ concentrations below 10 mmol/L. In contrast, a slight inhibitory effect was noted for para-aminobenzoic acid (ACT) at 10–20 mmol/L Cl^−^ [71], and a more pronounced negative impact was demonstrated on SMX degradation in an Fe^2+^-zeolite/PAA system [58]. This evidence underscored that the effect of Cl^−^ cannot be generalized and must be evaluated for each specific PAA-based AOP and pollutant combination.

##### Phosphate

The activation of asymmetric peroxides by inorganic anions through nucleophilic attack represents an important non-radical pathway. Yang et al. [72] used various anions including SO_4_^2−^, NO_3_^−^, and HPO_4_^2−^ to activate PMS, H_2_O_2_, and PS for degrading acid orange 7 (AO7). It was found that only HPO_4_^2−^ could effectively activate PMS, which was attributed to the asymmetric structure of PMS facilitating a nucleophilic attack by phosphate anions, potentially generating hydroxyl and sulfate radicals as reactive species. These findings were corroborated by Lou et al. [73], where phosphate was shown to selectively activate PMS, but not other peroxides. A similar activation mechanism was observed for PAA in phosphate-buffered systems. Through radical quenching experiments and EPR analysis, Duan et al. [74] demonstrated that phosphate effectively activated PAA under neutral pH conditions, achieving the optimal degradation efficiency of DCF. The proposed mechanism involved a nucleophilic attack by phosphate anions on the O-O bond of PAA, leading to the generation of ·OH and organic radicals including CH_3_C(O)O· and CH_3_C(O)OO·, which were identified as the primary reactive species responsible for pollutant degradation.

### 3.3. Heterogeneous Catalytic Oxidation

Following the discussion on homogeneous activation, which often faces challenges such as metal leaching, secondary pollution, and difficulty in catalyst recovery, heterogeneous catalysis has emerged as a promising alternative for PAA activation [57,75]. In heterogeneous systems, solid catalysts (e.g., metal oxides, carbon-based materials) facilitate the cleavage of the PAA O–O bond through surface-mediated reactions, while allowing for easy separation, reuse, and often greater stability under varied water matrix conditions [76,77]. This approach not only mitigates the risks of metal ion release but also enables the design of catalysts with tailored active sites, enhancing both activity and selectivity towards target pollutants [23,77].

#### 3.3.1. Metal Catalyst

##### Nano CuO

**Figure 9:**
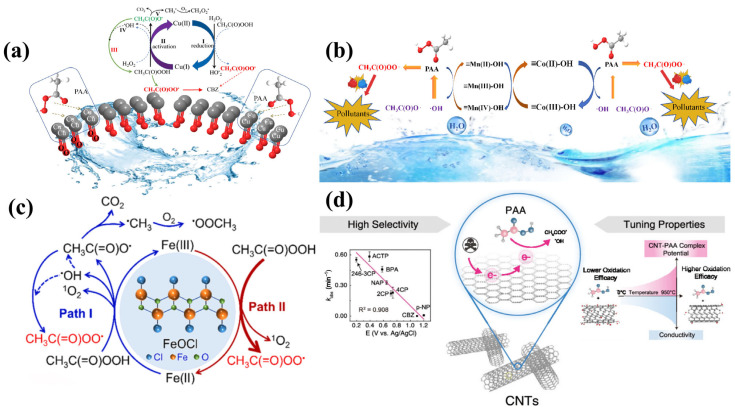
(**a**) Reaction mechanism of the nCuO/PAA system [77]; (**b**) possible mechanism of PAA activation by Co_1.9_Mn_1.1_O_4_ catalyst [87]; (**c**) PAA activation mechanism of FeOCl for SMX degradation in FeOCl/PAA system [89]; (**d**) reaction mechanism of CNT/PAA system [100].

Zhang et al. [77] investigated the degradation of CBZ using a nano-copper oxide (nCuO)-activated PAA system. The study revealed that under neutral pH conditions, nCuO initially formed a surface Cu(II)–peroxide complex upon contact with PAA. This interaction promoted electron transfer, leading to the reduction of Cu(II) to Cu(I) and the generation of CH_3_C(O)O· radicals. The continuous redox cycling between Cu(II) and Cu(I) was identified as crucial for sustaining the production of reactive species. As shown in Figure 9a, this cycle was further facilitated by H_2_O_2_ typically present in PAA solutions, which participated in the formation of additional Cu(II)-peroxide complexes that enhanced electron transfer (Path I) [78]. The catalytic decomposition of PAA by Cu(I) in situ generates a suite of radicals, including hydroxyl radicals (HO·) and CH_3_C(O)O·, while also reforming Cu(II)-OH complexes (Path II). Subsequently, CH_3_C(O)O· could react with residual PAA to yield acetyl peroxy radicals (CH_3_C(O)OO·) (Path III). The results of quenching experiments confirmed that CH_3_C(O)OO· was the main ROS responsible for CBZ degradation in the nCuO/PAA system [77]. The specific activation process is shown in (Equations (45)–(51)).

CH_3_C(O)OOH + Cu(II) → CH_3_C(O)OO-Cu(II) + H^+^ (45)

CH_3_C(O)OH + Cu(II) → Cu(I) + CH_3_C(O)OO· + H^+^ (46)

CH_3_C(O)OOH + Cu(I) → CH_3_C(O)O· + Cu(II)-OH^−^ (47)

CH_3_C(O)OOH + Cu(I) → Cu(II) + CH_3_C(O)O^−^ + HO· (48)

Cu(II) + H_2_O_2_ → Cu(I) + HO_2_·+ H^+^ (49)

Cu(I) + H_2_O_2_ → Cu(II) + HO· + OH^−^ (50)

CH_3_C(O)OOH + CH_3_C(O)O· → CH_3_C(O)OO· + CH_3_C(O)OH (51)

The degradation efficiency of CBZ in the nCuO/PAA process exhibited a strong pH dependence, with optimal performance observed under neutral conditions. The catalytic activity was compromised in both acidic and alkaline environments through distinct deactivation pathways. Under alkaline conditions, the nCuO surface became negatively charged, creating electrostatic repulsion with the anionic PAA species (PAA^−^) and impeding the formation of the essential catalyst–oxidant complex. Furthermore, OH^−^ ions promoted the precipitation of inactive copper hydroxide species on the catalyst surface. In acidic media, although the neutral PAA species predominates, its stronger O–O bond rendered it more difficult to activate [33]. Low pH also accelerated the corrosive leaching of Cu ions from nCuO, leading to irreversible catalyst deactivation. Moreover, the presence of carbonate and bicarbonate anions significantly inhibited degradation by scavenging ROS, including CH_3_C(O)OO· and HO·, thereby suppressing the overall oxidative capacity of the system.

##### Co-Mn Spinel Oxides

The catalytic activation of PAA by transition metals has attracted considerable interest as an efficient AOP. Transition metals such as Co, Mn, and Fe activate the O–O bond of PAA through electron transfer, generating highly reactive organic radicals (e.g., CH_3_C(O)O·, CH_3_C(O)OO·) without external energy input [79,80,81]. Among these, cobalt-based catalysts demonstrated superior activity. However, homogeneous Co^2+^ systems suffered from metal leaching and secondary pollution, limiting their practical application. To address this, recent research has shifted toward heterogeneous catalysts. Hu et al. [82] highlighted that cobalt-based heterogeneous materials, particularly bimetallic oxides, exhibited high stability and low Co ion release. Spinel-type oxides (AB_2_O_4_), especially those incorporating Co and Mn [26], have been widely studied due to their cost-effectiveness, ease of synthesis, magnetic separability, and structural stability, which effectively suppress metal leaching [83]. CoFe_2_O_4_ has shown potential in activating PAA, but its application was hindered by high catalyst dosage requirements [84]. It was demonstrated that introducing Mn into cobalt spinel structures significantly enhanced Fenton-like catalytic activity [85]. This is because the multi-valent redox cycling of Mn^2+^/Mn^3+^/Mn^4+^, which promoted electron transfer and facilitated PAA decomposition [86]. Zhang et al. [87] developed a series of Co_3−x_Mn_x_O_4_ catalysts, among which Co_1.1_Mn_1.9_O_4_ achieved nearly complete removal of SMX within 7 min. Quenching experiments and ESR analyses identified CH_3_C(O)OO· as the main oxidative species responsible for SMX degradation.

The degradation mechanism of SMX is illustrated in Figure 9b. Surface Co(II)–OH and Mn(II)–OH species were formed via hydrolysis at Lewis acid sites. PAA was adsorbed through hydrogen bonding, where electrons transferred from metal–OH to PAA generated CH_3_C(O)O· and HO-. The resulting CH_3_C(O)O· could further react with PAA to yield CH_3_C(O)OO·, while electron donation from PAA to high-valence Mn(III)–OH species regenerated the active sites, as detailed in (Equations (52)–(59)) [87].

CH_3_C(O)OOH + Co(II)-OH^−^ → Co(III)-OH^−^ + CH_3_C(O)O· + H^+^ (52)

CH_3_C(O)OOH + Co(II)-OH^−^ → Co(III)-OH^−^ + CH_3_C(O)O^−^ + HO· (53)

CH_3_C(O)OOH + Mn(II)-OH^−^ → Mn(III)-OH^−^ + CH_3_C(O)O· + OH^−^ (54)

CH_3_C(O)OOH + Mn(II)-OH^−^ → Mn(III)-OH^−^ + CH_3_C(O)O^−^ + HO· (55)

Mn(III)-OH^−^ + CH_3_C(O)OOH → Mn(IV)-OH^−^ + CH_3_C(O)O· + OH^−^ (56)

Co(III)-OH^−^ + CH_3_C(O)OOH → Co(II)-OH^−^ + CH_3_C(O)OO· + H^+^ (57)

Mn(III)-OH^−^ + CH_3_C(O)OOH → Mn(II)-OH^−^ + CH_3_C(O)OO· + H^+^ (58)

Mn(IV)-OH^−^ + CH_3_C(O)OOH → Mn(III)-OH^−^ + CH_3_C(O)OO· + H^+^ (59)

##### FeOCl

As a representative heterogeneous iron-based catalyst, FeOCl possesses a characteristic layered or thorhombic structure, where interlayers are connected by weak van der Waals forces via chlorine atoms [88,89]. The outermost Cl atoms are particularly susceptible to substitution, while the linear Cl–Fe–O and Fe–O–Fe framework exposes unsaturated iron atoms on the surface [90]. This unique layered configuration facilitates electron transfer and promotes the reduction of Fe (III) to active Fe (II), contributing to its superior catalytic performance compared to conventional iron-based catalysts [89,90]. Notably, FeOCl has been reported to exhibit activation efficiency up to 1000 times higher than other iron-based materials in certain catalytic processes [91]. Cheng et al. [89] evaluated the performance of an FeOCl/PAA system for SMX degradation. Under neutral conditions, a removal efficiency of 90.37% was achieved with a rate constant of 0.0836 min^−1^. Quenching experiments, ESR spectroscopy, and LC-MS analysis identified that CH_3_C(O)OO· radicals were the dominant ROS responsible for SMX breakdown; the specific activation process is shown in (Equations (45)–(51)).

CH_3_C(O)OOH + Fe(II) → CH_3_C(O)O· + Fe(III) + OH^−^ (60)

CH_3_C(O)OOH + Fe(II) → CH_3_C(O)O^−^ + Fe(II) + ·OH (61)

CH_3_C(O)OOH + Fe(III) → CH_3_C(O)OO· + Fe(II) + H^+^ (62)

CH_3_C(O)OOH + CH_3_C(O)OO^−^ → CH_3_C(O)O^−^ + CH_3_C(O)OH + ^1^O_2_ (63)

##### Mn_3_O_4_

Manganese oxide, particularly in the form of hausmannite (Mn_3_O_4_), has gained recognition as a common catalyst due to its low cost, natural abundance, and environmental compatibility [92]. Mn_3_O_4_ possesses a stable spinel structure with the formula [Mn^2+^(Mn^3+^)]_2_O_4_. It was synthesized by Saket Osgouei et al. [93] via a low-temperature hydrothermal and precipitation method. In this process, MnCl_2_·4H_2_O was reacted with sodium hydroxide to form a white Mn(OH)_2_ precipitate (Equation (64)), which was subsequently partially oxidized by dissolved oxygen to brown MnO(OH)_2_ (Equation (65)). Redox and dehydration reactions between these intermediates (Equations (66) and (67)), followed by Ostwald ripening and isotropic growth, yielded uniformly sized Mn_3_O_4_ nanoparticles [93,94,95].

MnCl_2_ + 2NaOH → Mn(OH)_2_ + 2NaCl (64)

2Mn(OH)_2_ + O_2_ → 2MnO(OH)_2_ (65)

2Mn(OH)_2_ + MnO(OH)_2_ → Mn_3_O_4_ + 3H_2_O (66)

6Mn(OH)_2_ + O_2_ → 2Mn_3_O_4_ + 6H_2_O (67)

SMX could be completely degraded at a catalyst dosage of 50 mg/L and pH 6.5 in the Mn_3_O_4_/PAA system. The catalytic mechanism involved the oxidation of Mn(II) and Mn(III) to higher valent states, coupled with the concurrent reduction of the Mn species, establishing continuous redox cycles between Mn(II)/Mn(III) and Mn(III)/Mn(IV). The results of radical quenching experiments, EPR, and LC-MS analysis confirmed that CH_3_C(O)O· and CH_3_C(O)OO· radicals were the key reactive species responsible for SMX degradation [93].

##### CoFe_2_O_4_

Heterogeneous cobalt-based catalysts have been explored to address the limitations of homogeneous Co^2+^/PAA systems, particularly cobalt ion residue and secondary pollution. CoFe_2_O_4_, known for its catalytic activity in persulfate activation, was evaluated for PAA activation under neutral conditions using SMX as the target pollutant [10,54]. The SMX removal efficiency of 87.29% was achieved under the PAA concentration of 200 μmol/L and catalyst dosage of 0.1 g/L. The degradation was primarily attributed to the redox cycling between Co^3+^/Co^2+^ on the catalyst surface, activating PAA to generate organic radicals (RO·) responsible for SMX degradation. Furthermore, the magnetic properties of CoFe_2_O_4_ facilitate easy separation and recovery, highlighting its potential for practical applications [96].

##### Co_3_O_4_

Transition metals such as iron and cobalt are well-documented for their effectiveness in activating peroxides like H_2_O_2_ and PMS, and generating radicals that efficiently degrade pollutants [97,98,99]. However, homogeneous catalytic systems often entail risks of metal leaching and secondary contamination [62]. To overcome these issues, Wu et al. [75] developed a heterogeneous Co_3_O_4_/PAA system for degrading orange G (OG) under neutral conditions. Complete degradation of OG was achieved within 90 min, with negligible cobalt leaching observed. Mechanistic studies confirmed that organic radicals, specifically CH_3_C(O)O· and CH_3_C(O)OO·, were the dominant reactive species responsible for pollutant degradation.

#### 3.3.2. Non-Metallic Catalysts

##### Activated Carbon (AC)

A calcination modified activated carbon (AC600) was developed by Dai et al. [76] through calcination of commercial activated carbon at 600 °C, without atomic doping or metal loading. The AC600/PAA system was applied for SMX removal in groundwater under mild contamination conditions, achieving 99.4% degradation within 150 min. Degradation proceeded via both radical and non-radical pathways: PAA was activated by AC600 to generate HO·, CH_3_C(O)O·, and CH_3_C(O)OO· radicals (Equations (68)–(71)), while electron transfer and dissolved oxygen played a negligible role. Optimal performance was observed between pH 3–9 using 50 mg/L AC600 and 0.26 mM PAA. Cl^−^ ions slightly enhanced SMX removal, whereas HCO_3_^−^ exhibited strong inhibition. The AC600/PAA system offers an eco-friendly and cost-effective strategy for groundwater remediation with minimal secondary pollution risk.

AC-e^−^ + CH_3_C(O)OOH → CH_3_C(O)O· + OH^−^ (68)

AC-C=O^−^ + CH_3_C(O)OOH → AC-C=O + CH_3_C(O)O· + OH^−^ (69)

AC-C=OH^−^ + CH_3_C(O)OOH → AC-C-O^−^· + CH_3_C(O)O· + H_2_O (70)

AC-C=OOH^−^ + CH_3_C(O)OOH → AC-C-OO^−^· + CH_3_C(O)O· + H_2_O (71)

##### Graphene

Graphene and its derivatives have attracted significant attention as metal-free catalysts for PAA activation, owing to their unique two-dimensional structure, high specific surface area, and exceptional electronic conductivity. Since its successful isolation, graphene has been exhibiting outstanding mechanical, thermal, optical, and electrical properties [101,102]. These characteristics enable graphene-based materials to activate PAA through surface defects, functional groups, or heteroatom-doped sites, generating organic oxygen radicals such as CH_3_C(O)O· and CH_3_C(O)OO· as the key reactive species [103]. Sun et al. [103] demonstrated that nitrogen-doped graphene (N-G) could activate PAA to degrade SMX, achieving over 95% removal within 3 min under neutral conditions, with CH_3_C(O)O· identified as the dominant radical. Yuan et al. [104] reported that reduced graphene oxide (rGO) activated PAA for efficient BPA degradation, achieving near complete removal within 15 min across a broad pH range, where both HO· and CH_3_C(O)O· were verified as key active species. These studies highlighted the versatility and high reactivity of GO-based catalysts in PAA activation systems. The catalytic performance of various graphene materials, including GO and rGO, and nitrogen-doped graphene (N-G), has been systematically compared in Table 4, which summarizes their active sites, dominant radical species, and operational advantages and limitations. This comparison provides valuable guidance for the rational design and optimization of carbon-based catalysts in AOP [105].

##### Carbon Nanotubes (CNTs)

Carbon nanotubes (CNTs), characterized by low surface functionality and high sp^2^-hybridized carbon content, activate PAA through mechanisms distinct from those of activated carbon. Zhang et al. [106] investigated CNT/PAA systems for phenolic pollutant degradation and identified, through experiments and density functional theory (DFT) calculations, that electron transfer occurs primarily at the sp^2^-carbon domains, leading to the formation of a metastable CNT–PAA complex. This complex subsequently decomposes to generate CH_3_C(O)O· and HO·, though the latter plays a minor role. Some of these complexes then decompose to generate HO and CH_3_C(O), as shown in (Equations (72) and (73)).

CNT—OH + CH_3_C(O)OOH → HO· + CH_3_C(O)O^−^ + CNT = O + H^+^ (72)

CNT—OH + CH_3_C(O)OOH → CH_3_C(O)O· + CNT = O + H_2_O (73)

Zhang et al. [106] reported that CNT activation enhanced BPA removal from 5% with PAA alone to 96.4%, confirming the effective generation of reactive species. Kong et al. [100] systematically modified commercial CNTs to vary their conductivity, specific surface area, defect density, oxygen content, and graphitization degree. As illustrated in Figure 9d, pollutant degradation efficiency was positively correlated with the oxidation potential of the CNT–PAA complex and CNT’s electrical conductivity. Higher specific surface area, lower oxygen content, and increased graphitization were found to enhance activation performance. Carbon-based materials such as AC, graphene, and CNTs offer structural tunability and wide availability, making them promising catalysts for PAA activation.

## 4. Applications of Activated PAA

### 4.1. Application in Wastewater

#### 4.1.1. Degradation of Organic Pollutants

Peracetic acid (PAA) is widely used as a disinfectant and oxidizer in various industries, including wastewater treatment. The reaction kinetics and transformation pathways of several β-lactam antibiotics under PAA treatment have been systematically studied, highlighting its efficacy in degrading pharmaceuticals commonly detected in effluents and surface waters [107]. In recent years, PAA-based AOPs have gained attention as a promising strategy for water purification [46]. Upon activation, the peroxide bond (O–O) in PAA undergoes homolytic cleavage, generating highly reactive species such as hydroxyl radicals (HO·) and organic radicals (e.g., CH_3_C(O)O·, CH_3_C(O)OO·). These radicals enable efficient degradation of diverse organic contaminants, including pharmaceuticals [32,37,58,108,109], phenols [48,110], and dyes [111]. The performance of various PAA activation systems in treating these pollutants is summarized in Table 5, providing a reference for selecting suitable techniques under specific wastewater conditions.

#### 4.1.2. Disinfection and Sterilization

PAA is a potent peroxycarboxylic acid oxidant widely employed as a disinfectant in food safety, healthcare, and wastewater treatment due to its strong microbial inactivation capacity and minimal formation of harmful byproducts [25]. Global consumption of PAA reached approximately 170,000 tons in 2013, reflecting its broad applicability [25]. The inherent instability of the peroxide bond allows PAA to decompose into reactive species, enabling its dual use as both a disinfectant and an oxidant for organic pollutant degradation. In wastewater treatment, PAA has demonstrated effective disinfection performance. Koivunen et al. [112] reported that a dose of 2–7 mg/L with 27 min of contact time achieved a 3-log reduction in total coliform and enterococci in secondary and tertiary effluents. Dunkin et al. [113] demonstrated that 41.8 mg/L and 2.3 mg/L were needed for 1-log reduction in MS2 phage and murine norovirus in secondary effluent, respectively. Beyond disinfection, PAA is also used in cooling towers, membrane cleaning, combined sewer overflow treatment, and biosolid sterilization [25,114].

### 4.2. Application in Groundwater

In groundwater remediation, PAA-based AOPs have shown significant potential for in situ chemical oxidation. For instance, Lin et al. [115] developed an ABTS/Fe(II)/PAA system in which ABTS served as an electron shuttle to promote Fe(III)/Fe(II) cycling, significantly enhancing diclofenac degradation under acidic conditions, with ABTS^+^ identified as the primary oxidizing species. Zhao et al. [116] designed a Cu@NCs catalyst to activate PAA for sulfadiazine (SMT) degradation in groundwater at neutral pH. The system achieved 94.5% SMT removal efficiency within 30 min using only 50.0 μM PAA, with both radical (R–O·) and non-radical (^1^O_2_) pathways contributing to the degradation. Similarly, Dai et al. [76] used thermally modified activated carbon to activate PAA for SMX degradation in groundwater, where organic radicals (CH_3_C(O)OO· and CH_3_C(O)O·) and direct electron transfer were identified as key mechanisms.

Despite these promising results, the practical application of PAA-AOPs in groundwater is challenged by complex aquifer matrices. Natural organic matter, carbonates, and heavy metal ions can scavenge reactive species, reducing the degradation efficiency of target pollutants. Future studies should focus on developing matrix-resistant catalytic systems and optimizing operational conditions to improve the efficacy and reliability of PAA-based technologies for groundwater remediation.

### 4.3. Application of Activated PAA in Soil

PAA-based AOPs have emerged as a promising technology for remediating soils and sediments contaminated with persistent organic pollutants such as polycyclic aromatic hydrocarbons (PAHs), many of which are recognized carcinogens [117,118]. Due to their high hydrophobicity, PAHs tend to accumulate in sediments, posing long-term environmental risks. While various oxidation technologies have been explored, kinetic limitations often hinder their efficiency. PAA exhibits a high redox potential and greater hydrophobicity than hydrogen peroxide, facilitating its access to adsorbed contaminants. When activated through advanced oxidation processes, PAA generates a reactive species that enhance degradation kinetics without producing persistent toxic byproducts, as it decomposes into H_2_O, CO_2_, and acetic acid [119].

As summarized in Table 6, activated PAA systems achieved high removal efficiencies (≥90%) for pollutants such as α-methylnaphthalene and benzocaine in sediments under near-neutral pH within 24 h [120]. However, removal efficiency was influenced by sediment properties. Specifically, high organic carbon content could sequester pollutants but also compete for reactive species, while particle size and surface area affect oxidant–pollutant contact. Site-specific factors thus play a critical role, necessitating preliminary geological assessments for effective application. Future studies should focus on developing tailored activation strategies to enhance the adaptability and efficiency of PAA-based remediation in complex real-world environments.

## 5. Challenges of Activated PAA Advanced Oxidation Technology

### 5.1. Consensus and Challenges in the Identification of Reactive Oxygen Species

Clarifying the dominant active oxygen species (ROS) in different PAA activation systems is the key to understanding their oxidation properties and selectivity. However, the accurate identification of ROS is often uncertain due to the short life of the species, the interference of the detection method, and the complexity of the water matrix, which often lead to contradictions or excessive simplification in the literature. The author systematically sorts out the consensus and controversies in the current cognition. Table S1 summarizes the main ROS widely accepted under different activation systems, relevant experimental evidence, major academic controversies, and secondary species that may be ignored, providing a clearer mechanism identification framework for future research.

### 5.2. Toxicity Risks of Transformed Products and Operational Safety Challenges of PAA

(PAA)-based AOPs exhibit a dual nature in terms of byproduct formation. A significant advantage lies in the environmentally benign decomposition pathway of PAA, which yields only acetic acid, water, and oxygen as end products, presenting a clear advantage over chlorination and persulfate-based processes. However, potential risks are associated with the activation process. The use of homogeneous metal activators carries the risk of metal ion leaching and subsequent secondary pollution [87]; furthermore, in bromide-containing waters, PAA and its derived radicals can promote the oxidation of bromide to carcinogenic bromate [124]. Consequently, the degradation of organic pollutants by PAA-AOPs is not always a harmless path to complete mineralization. The environmental risks of its transformation products (such as toxicity and persistence) may be completely different from that of original pollutants, and even induce new risks in halogen-containing substrates. As shown in Table S3, the system compares the potential differences in key environmental risk properties between PAA-induced transformation products and original emerging pollutants [125].

In addition to the environmental toxicity of transformation products, the practical application of PAA-AOP technology must also consider the occupational health and safety risks of oxidants themselves in the process of production, storage, transportation, and addition. Compared with common AOP oxidizing agents such as hydrogen peroxide (H_2_O_2_) and persulfate (PMS/PDS), high-concentration PAA poses a more severe and unique management challenge due to its inherent instability, strong volatility, and high corrosiveness. To clarify this difference for the system, as shown in Table S4, the core risk characteristics of high concentrations of PAA and H_2_O_2_ and PMS/PDS are compared in terms of physical hazards, health hazards, storage requirements, and emergency response. The comparison shows that the use of PAA is often accompanied by higher safety protection costs and stricter operational management requirements, which are key factors that must be taken into account in technical and economic analysis and engineering design.

### 5.3. Economic Cost and Operational Feasibility

While the unit cost of PAA is higher than that of H_2_O_2_, a holistic assessment of activated PAA systems must be conducted [31]. Although heterogeneous catalysts or energy activation require higher initial investment due to complex synthesis or energy consumption, they can substantially lower the required PAA dosage and avoid costs associated with metal sludge handling [126,127,128,129]. Moreover, most PAA-AOPs operate effectively at neutral pH, eliminating the need for frequent pH adjustment common in traditional Fenton processes, thereby simplifying operation and reducing chemical costs.

There are significant differences in energy consumption and the cost of different activation methods. For example, the equipment investment and power consumption of ultraviolet activation (UV/PAA) mainly come from ultraviolet lamps; the energy consumption of electrochemical activation (EC/PAA) is directly related to current density and reaction time. Some studies show that under optimized conditions, the overall processing cost of the activated PAA system may be comparable to or even more advantageous than that of traditional processes due to its fast reaction rate and wide pH adaptation range [29,126].

Transitioning PAA-based AOPs from laboratories to real-world implementation requires overcoming significant challenges posed by complex environmental matrices. Key issues include radical scavenging by background constituents, limited oxidant diffusion in heterogeneous media, and the risk of hazardous byproduct formation. Table S2 provides a comparative summary of these core challenges across different application fields (wastewater, groundwater, etc.) and outlines potential countermeasures informed by current research [70,126,130].

At the practical operation level, storage stability (easy to decompose), corrosiveness, and some activation technologies (such as homogeneous metal ion activation) may bring secondary pollution risks of PAA. In contrast, although the non-homogeneous catalytic system is easy to recycle, it also faces long-term operation and maintenance problems such as catalyst inactivation, regeneration, and fixed bed blockage. Energy activation (such as UV and US) depends on special equipment, and the energy efficiency and reliability of its large-scale application need to be further verified. Table 7 provides simplified comparison data of several activation systems based on typical scenarios, and clarifies their applicable boundary conditions [84,127,128,129,130].

In summary, the PAA activation technology offers a versatile platform for organic pollutant degradation. Homogeneous activation is characterized by process simplicity but demands precise dosage control and involves risks in handling corrosive chemicals [25,114,131,132]. Heterogeneous systems enable easier catalyst recovery and are suitable for continuous-flow reactors, though long-term challenges such as catalyst fouling and deactivation remain [125,126,133,134]. Direct energy activation facilitates automation but entails higher capital and operational energy costs [126]. Therefore, the selection of an appropriate activation strategy should be guided by specific water matrix conditions, treatment objectives, and economic considerations.

## 6. Conclusions and Prospects

This review has systematically analyzed PAA activation as an advanced oxidation process (AOP) for degrading organic pollutants. Key mechanisms, activation methods (such as UV, metal ions, carbon-based catalysts), and applications in wastewater, groundwater, and soil remediation have been summarized. Environmental performance and operational feasibility were also evaluated. PAA generates reactive species including ·OH, CH_3_C(O)O·, and CH_3_C(O)OO· under various activation conditions, enabling the efficient removal of pharmaceuticals, phenols, and dyes. However, the practical application of PAA-based AOPs still faces several key challenges, which also represent important opportunities for future research.

First, the mechanisms of non-radical pathways in PAA activation require further clarification. Although studies have confirmed the dominant roles of organic radicals (e.g., CH_3_C(O)OO·) and singlet oxygen (^1^O_2_) in certain systems, their generation mechanisms, reaction selectivity, and transformation pathways in complex water matrices remain poorly understood. In particular, the competition between radical and non-radical pathways in the presence of common anions such as phosphate and chloride lacks systematic investigation. Combining in situ spectroscopic techniques (e.g., advanced EPR) with theoretical calculations (e.g., DFT) could help elucidate the kinetics and pathways of non-radical species, providing a theoretical basis for their directed regulation under realistic water conditions.

Second, the development of low-leaching and highly stable cobalt-based catalysts remains insufficient. Although Co^2+^ is one of the most efficient homogeneous activators of PAA, its biotoxicity and potential for environmental accumulation limit practical application. Heterogeneous catalysts such as CoFe_2_O_4_ and Co_3_O_4_ have been explored, but they still face challenges of cobalt leaching and activity loss during long-term operation. Future work should focus on designing tailored structures such as bimetallic synergistic catalysts (e.g., Co–Mn spinels) and enhancing catalyst stability and recyclability through surface modification and structural engineering.

Third, more attention should be paid to degradation pathways, intermediate products, and ecological toxicity. Most current studies emphasize removal efficiency, while the identification of transformation products and their toxicological implications is often overlooked. This gap is particularly concerning for chloride- or bromide-containing water, where PAA-based systems may form halogenated byproducts (e.g., bromate, chlorinated organics), introducing additional environmental risks. Future research should integrate high-resolution mass spectrometry (LC-HRMS) with toxicity assays (e.g., acute/chronic and genotoxicity tests) to systematically identify critical transformation products and support environmental safety assessments of PAA-AOPs.

Moreover, while existing research has focused largely on pharmaceuticals and personal care products, future studies should expand to other refractory pollutants, such as pesticides and poly-/perfluoroalkyl substances (PFASs), to better evaluate the broad applicability of PAA-based oxidation.

Finally, more techno-economic assessments and long-term stability studies are essential to facilitate the scaling-up of PAA-based processes from laboratory-scale research to full-scale industrial implementation.

## Figures and Tables

**Table 1:** Comparison of physicochemical properties of major oxidants.

Property	PAA	H_2_O_2_	PDS	PMS
Molar mass (g/mol)	76.05	34.01	270.33	614.76
Density (kg/L)	1.04	1.71	2.48	1.20
Melting point (°C)	0.1	−0.43	100	100
Boiling point (°C)	105	150	-	-
Flash point (°C)	41	107	-	-
Acidity (pKa)	8.2	11.75	2.5	9.4
Stability	Unstable	Moderate	Moderate	Moderate
Solubility	Soluble in water, ethanol, and sulfuric acid	Soluble in water, ethanol, ether	Soluble in water	Soluble in water
Redox potential (V)	1.96	1.78	2.10	1.82
O-O Bond energy (kJ/mol)	160.0	213.4	140	145

**Table 2:** Comparison of the performance of degrading organic pollutants by different PAA direct activation methods.

Degraded Organic Pollutants		PAA Potency (µmol/L)	Type and Dosage of Activator		Main ROS	Removal Efficiency	Advantages	Disadvantages	Ref.
Target	Pollutant Concentration (µmol/L)		System	Dose					
SMX	5.0	100	MW	Microwave output power 500 W, reaction temperature 60 °C, pH = 8.0	^1^O_2_, CH_3_C(O)O·, CH_3_C(O)OO·	94.2%	Fast, uniform energy transfer	The cost is high, and the energy efficiency depends on the absorption capacity of microwaves by the reaction system, which makes it difficult in the practical application	[27]
SFX	19.7	660	UV	Ultraviolet wavelength λ = 254 nm, intensity 0.65~3.50 kW/m^3^, pH = 7	·OH, CH_3_CO_2_	95.0%	Easy to operate, scalability	The penetration ability of ultraviolet light in water is limited and the energy consumption is high	[45]
TCH	2.1	130	US	Ultrasonic power 1625 W/L, pH = 7	^1^O_2_, CH_3_C(O)OO·	99.4%	High efficiency	There are problems in the mass transfer limitation and uneven distribution of sound energy for large-scale application	[34]
SMX	5.0	200	heat	Reaction temperature 60 °C, pH = 7	^1^O_2_, HO·, CH_3_C(O)O·, CH_3_C(O)OO·	86.0%	Simple operation, low cost	High energy consumption	[37]
MB	31.3	3600	EC	The positive extreme platinum sheet (Pt); the negative extreme graphite plate, current density 10 mA/cm, pH = 3.0	HO·, CH_3_(O)O·, CH_3_C(O)OO·	93.9%	No additional reagents, no secondary pollution	Electrodes are easy to wear and high operating cost	[22]

**Table 3:** Degradation performance and main reactive species in homogeneous PAA activation systems.

Degraded Organic Pollutants		PAA Potency (µmol/L)	Type and Dosage of Activator		Main ROS	Removal Efficiency	Advantages	Disadvantages	Ref.
Target	Pollutant Concentration (µmol/L)		System	Reaction Conditions					
TMP	5.0	1140	Cr(III)/PAA	[PAA]_0_: [Cr(III)]_0_ = 5:1(PAA = 1315 μM, Cr(III) = 263 μM), pH 8.0	HO·, CH_3_C(O)O·	90%	Cr(III) can activate PAA to produce · OH and high-valent chromium species (Cr(IV)/Cr(V)).	Cr(VI) (carcinogen) will be generated during the reaction process.	[51]
NPX	15	100	Fe(II)/PAA	[PAA]_0_: [Fe(II)]_0_ = 1:1 (PAA = 100 μM, Fe(II) = 100 μM), pH 3.0	CH_3_C(O)O·, HO·	98.2%	Fe(II) has low toxicity, a wide range of sources, and produces ·OH, carbon-centered free radicals and Fe(IV), forming a multipath oxidation mechanism.	Fe(II) is easily oxidized into Fe(III) in the air, affecting its activity and durability.	[46]
SMX	10	100	Co(II)/PAA	[PAA]_0_ = 100 μM, [Co]_0_ = 0.8 μM, pH 7.0	CH_3_C(O) O·, CH_3_C(O) OO·	80%	An extremely low amount of cobalt can be activated efficiently; Co^3+^/Co^2+^ efficient circulation, insensitive to chloride ions	Cobalt has certain risks of biological toxicity and environmental accumulation.	[47]
CBZ	10	200	Mn(II)/PAA/EDTA	[PAA]_0_: [Mn(II)]_0_ = 4:1 (PAA = 200 μM, Mn(II) = 50 μM), pH 5.5, EDTA = 100 µM	HO·, CH_3_C(O)O·, CH_3_C(O)OO·	>80%	Mn(II) is naturally rich and low-cost.	If there is no ligand stability, Mn(III) will quickly differentiate into Mn(II) and MnO_2_.	[30]
SMX	10	200	Ru(III)/PAA	[PAA]_0_: [Fe(II)]_0_ = 2:1 (PAA = 200 μM, Ru(III) = 100 μM), pH 7.0	CH_3_(O)O·, CH_3_C(O)OO·	100%	Completely degrade SMX within 2 min; superior to Fe(II), Co(II), Mn(II), and anti-phosphate interference; overcome the limitations of Co(II) and other systems	Ru(III) is high-cost.	[67]

[PAA]_0_ and [Cr(III)]_0_ represent the initial concentration, respectively.

**Table 4:** Catalytic systems and mechanisms of graphene materials.

System	Target	Key Active Sites	Main ROS	Advantages	Limitations	Ref.
Nitrogen-doped graphene (N-G)	SMX	Nitrogen doping produces carbon defects and graphite nitrogen, which promotes electron transfer	CH_3_C(O)O·	No metal leaching, wide pH range, high activity	The synthetic cost is relatively high.	[103]
Reduced oxidized graphene (rGO)	Ibuprofen, diclofenac	Sp^2^ carbon network, residual carbonyl, and other functional groups	HO·, R-O·	Good electrical conductivity, strong electron transport capacity	It is easy to aggregate, and the active site may be unstable.	[104]
Graphene oxide (GO)	Dye molecules	Surface oxygen-containing functional groups (such as carboxyl groups)	HO·, R-O·	Simple to prepare, but usually low in activity	The catalytic activity is usually low.	[105]
Graphene–metal oxide composite	A variety of refractory organic matter	Conductive and dispersion carrier effects of graphene	HO·, R-O·	High activity and strong synergistic effect, but attention should be paid to metal leaching	There is a risk of metal leaching	[105]
Activated carbon (AC)	SMX	Rich surface oxygen-containing functional groups and pore structures	HO·, CH_3_C(O)O·	Larger surface area, low cost, rich functional groups	There is competitive adsorption, and the mass transfer resistance may be large.	[76]
Carbon nanotube	BPA	Sp^2^ carbon domain, surface defects and structure	CH_3_C(O)O·, HO·	Excellent electrical conductivity and good mass transfer performance	It is easy to reunite with van der Waals forces, and the cost is relatively high.	[100]

**Table 5:** Application of activated PAA technology to degrade organic pollutants in wastewater.

Degraded Organic Pollutants			PAA Potency (mmol/L)	Type and Dosage of Activator		Main ROS	Removal Efficiency	Ref.
Type	Target	Pollutant Concentration/(µmol/L)		System	Activator Dosage			
Fuel	MB	31.26	3.6	EC/PAA	The concentration of electrolyte Na_2_NO_3_ is 0.45 g/L; the current density is 10 mA/cm.	·OH, CH_3_C(O)O·, CH_3_C(O)OO·	93.99%	[22]
	Orange G	50	0.5	Co_3_O_4_/PAA	100 mg/L	CH_3_C(O)O·, CH_3_C(O)OO·	100%,	[54]
Phenolic organic matter	Phenol	10	0.1	CPANI/PAA	25 mg/L	^1^O_2_	96%	[110]
	Nitrophenol	143.9	5000	MV-MIL-53(Fe)/PAA	20 mg/L	·OH	100%	[48]
Medicines	OTC	≤10.86	0.066	UV/PAA	The wavelength is 254 nm, and the irradiation dose is 0~223.2 mJ/cm.	·OH	100%	[32]
	NOR	6.26	0.131	MPUV/PAA	The wavelength is 200~300 nm; the irradiation dose is 0~500 mJ/cm.	·OH, ^1^O_2_, ·O_2_·	96.6%	[108]
	SMT	35.93	0.1	UV/Fe0/PAA	The concentration of Fe^0^ is 0.1 g/L, the wavelength is 254 nm, and the power of the ultraviolet lamp is 6 W.	·OH, CH_3_C(O)O·, CH_3_C(O)OO·	85%	[109]
	SMX	5	0.2	heat/PAA	-	CH_3_C(O)O·, CH_3_C(O)OO·	86%	[37]
	SMX	10	0.55	CoFe_2_O_4_@Biomass charcoal/PAA	100 mg/L	CH_3_C(O)O·, CH_3_C(O)OO·	95.8%	[111]
	SMX	50	0.66	LaCoO_3_/PAA	20 mg/L	CH_3_C(O)O·, CH_3_C(O)OO·	100%	[10]
	SMX	5	400	Fe^2+^-Zeolite/PAA	800 mg/L	·OH	100%	[58]

**Table 6:** Application of activated PAA technology to degrade organic pollutants in soil sediment.

Degraded Organic Pollutants			Potency	Oxidizer Ratio	Physical Properties of Soil/Sediment	Factors Affecting the Removal Effect	Removing Effects	pH	Ref.
Type	Sediment Samples	Pollutants							
Lake sediment	Lake Macatawa (Holland, MI)	α methylnaphthalene	10~25 mmol/kg	The volume ratio of hydrogen peroxide, acetic acid, and deionized water is 1:1:1.	The total organic carbon content is 2.1~12.8%, and the specific surface area is 3.2 to 22.0 m^2^/g.	Organic carbon content and specific surface area of sediments	The removal rate of α-methylnaphthalene is 100% within 24 h.	7.49~7.67	[121]
Lake sediment	Sigma Aldrich Chemical	Benzo[a]pyrene	10~25 mmol/kg	The volume ratio of hydrogen peroxide, acetic acid, and deionized water is 1:1:1.	The total organic carbon content is 0.45~12.56%, and the specific surface area is 1.21 to 13.96 m^2^/g.	Organic carbon content and specific surface area of sediments	The removal rate of Benzo(a)pyrene is 100% within 24 h.	7.48~7.76	[122]
Superfund contaminated sites	Bedford LT lot 10 and Bedford LT soils	PAHs	The PAH concentrations of Bedford LT soils and Bedford LT lot 10 are 500~1000 and 2000~3000 mg/kg, respectively.	The volume ratio of hydrogen peroxide, acetic acid, and deionized aqueous solution is 3:5:7 or 3:3:9.	The water content of soils Bedford LT lot 10 and Bedford LT soils is 25% and 18.5%, and the total organic carbon content is 11% and 18.5%.	pH, total organic carbon content and particle size distribution	The 14 PAHs of Bedford LT were almost completely degraded within 24 h; the degradation of 14 PAHs was not observed in Bedford LT10.	7.04~7.09	[123]
Sandy and silty clay deposits	Lake Macatawa (Holland, MI) throughout the eastern basin	R-methylnaphthalene, benzo[a] naphthalene	500 mg/kg of R-methylnaphthalene or benzo[a]naphthalene	The volume ratio of hydrogen peroxide, acetic acid, and deionized water is 2:5:8.	The particle size of sand sediment is >150 µm; 150 µm > powdery clay particle size > 75 µm. Sand and powdery clay sediment samples contain about 0.5% and 1.4% of total organic carbon, respectively.	Sediment particle size and organic carbon content	24 h; the removal rate of α-methylnaphthalene is 90%; the removal rate of benzo[a]naphthalene is 90%.	7.08~7.12	[119]

**Table 7:** Comparison of Technical and Economic Parameters for Different Peracetic Acid Activation Methods.

The Way of Activation	Key Economic and Operational Parameters	Main Advantages	Main Challenges and Considerations	Refs.
Ultraviolet activation (UV/PAA)	Power consumption: ~0.1–0.5 kWh/m^3^ (depending on the UV dose). Equipment: UV reactor, lamp replacement. Applicability: suitable for low turbidity water bodies	Fast response, high degree of automation, no secondary residue	The light transmittance of water bodies is greatly affected; the life of the lamp is limited, and the cost of high turbidity water treatment has increased sharply.	[70,126]
Electrochemical activation (EC/PAA)	Power consumption: positive correlation with current density and time. Electrode cost: BDD electrodes are expensive, and graphite electrodes are cheaper. No need to add additional electrolytes (PAA itself can provide)	Active species are produced in situ, easy to integrate, and control automatically	The cost and life of electrode materials; high energy consumption; may produce halogen byproducts (if containing Cl^−^)	[22,127]
Non-homogeneous catalysis (such as Co_3_O_4_/PAA)	Catalyst cost: synthesis is complicated, but it can be reused. No continuous power consumption (after the reaction is started)	Wide range of pH applications; no potential metal-leaching risk	Catalyst recovery, inactivation and regeneration	[17,84,116]
Persulfate synergy (PAA/PS)	Oxidizer cost: PAA and PS costs need to be superimposed. There may be sulfuric acid residue.	Produce multiple free radicals, strong synergy and high degradation efficiency	The total cost of pharmaceuticals has increased, and the environmental impact of sulfate radical byproducts needs to be evaluated.	[130]

## Data Availability

No new data were created or analyzed in this study.

## Supplementary Materials

The following supporting information can be downloaded at: https://www.mdpi.com/article/10.3390/toxics14010006/s1, Figure S1. Molecular structures of polyacrylic acid (PAA) and hydrogen peroxide (H_2_O_2_). Figure S2. (a) The reaction mechanism of the Cr(III)/PAA system; (b) the Cr(III)/PAA system’s TMP degradation process is illustrated. Table S1. Consensus mechanism and controversy summary of reactive oxygen in PAA activation system. Table S2. Application challenges and countermeasures of activated peroxyacetic acid (PAA) technology in different environmental substrates. Table S3. Comparative analysis of PAA-induced transformation products and original ECs. Table S4. Comparison of occupational exposure and storage risks of high-concentration PAA and H_2_O_2_/PMS/PDS.

## Abbreviations

The following abbreviations are used in this manuscript:

PAA	Peroxyacetic acid
AOPs	Advanced oxidation processes
ECs	Emerging contaminants
DCF	Diclofenac
TMP	Trimethoprim
PBS	Phosphate-buffered solution
NPX	Naproxen
PDS	Peroxydisulfate
SMX	Sulfamethoxazole
MB	Methylene Blue
MMO	Mixed metal oxide
nCuO	Nano-copper oxide
OG	Orange glucose
N-G	Nitrogen-doped graphene
PMS	Peroxymonosulfuric acid
PPL	Propranolol
rGO	Reduced graphene oxide
GO	Graphene oxide
CNT	Carbon nanotubes
ABTS	2,2′-Azinobis(3-ethylbenzothiazoline-6-sulfonic acid) diammonium salt
PAHS	Polycyclic aromatic hydrocarbons
CBZ	Carbamazepine
ROS	Reactive oxygen species
NOM	Natural organic matter
TCH	Tetracycline hydrochloride
AO7	Acid Orange 7
PS	Polystyene
HP	Phosphoric acid
AC	Commercial activated carbon
BPA	bisphenol A
OTC	Oxytetracycline
NAP	Naproxen
