# Supplementary material for: Unlocking the Oxidative Performance of Peracetic Acid: A Comprehensive Review of Activation Pathways and Mechanisms for Environmental Remediation
Source: Toxics. 2025 Dec 19;14(1):6. doi: 10.3390/toxics14010006 (PMC12845966; doi:10.3390/toxics14010006)
Supplement: Supplementary file 1 [file toxics-14-00006-s001.zip › toxics-4005654-supplementary.pdf]

Supporting Information for

Unlocking the Oxidative Performance of Peracetic Acid: A Comprehensive Review of Activation Pathways and Mechanisms for Environmental Remediation

Chun Xiao [1,2,3], Lihong Ai [1], Jinxi Chen [1], Wu Ren [1], Jinran Feng [4], Yue Lu [1], Yaoyao Chen [1], Yunxiu Luo [1], Xindong Yang [1], Min Dai [1,2], Jiangfei Cao [1,2,3], Jianqiao Qin [1,2] and Chunsheng Xie [1,2,3,*]

1 College of Environmental and Chemical Engineering, Zhaoqing University, Zhaoqing 526061, China; 13554301718@163.com (C.X.)
2 Guangdong Provincial Key Laboratory of Eco-Environmental Studies and Low-Carbon Agriculture in Peri-Urban Arease, Zhaoqing University, Zhaoqing 526061, China
3 New Energy and New Materials Research Center, Zhaoqing University, Zhaoqing 526061, China
4 College of Mechanical and Automotive Engineering, Zhaoqing University, Zhaoqing 526061, China
* Correspondence: xiechsh@126.com

This supporting information include 2 figures (Figure S1 to S2) and 4 tables (Table S1 to S4)

Figures:

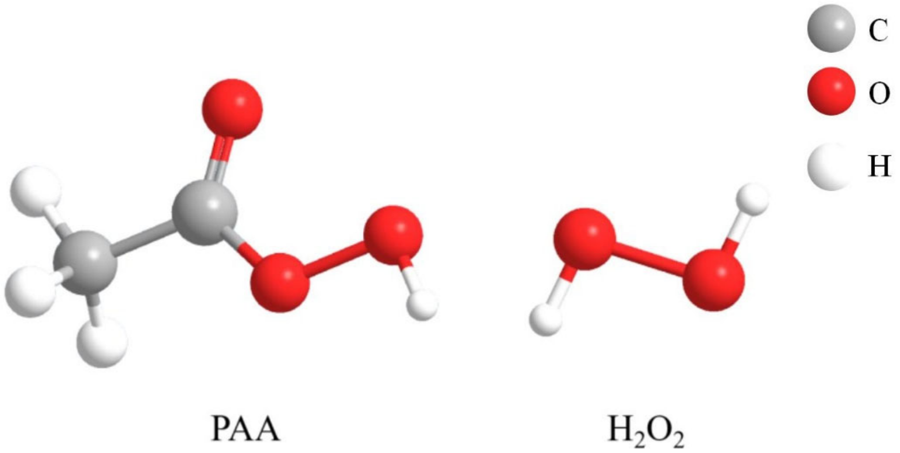

Figure. S1. Molecular Structures of Polyacrylic Acid (PAA) and Hydrogen Peroxide (H_2O_2)

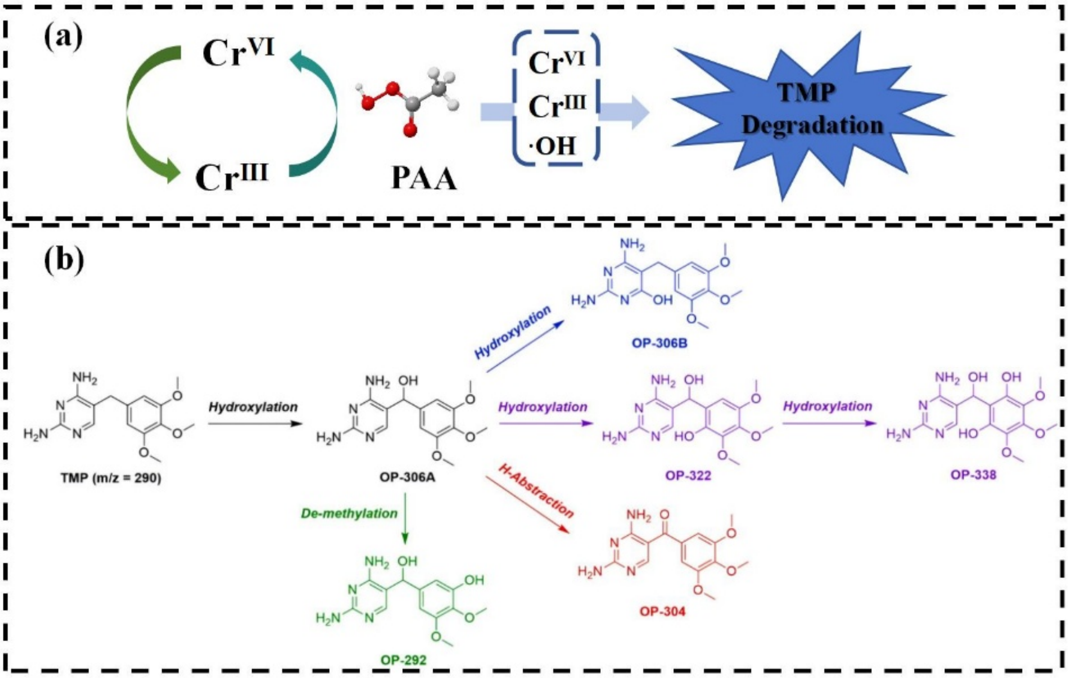

Figure. S2. (a) The reaction mechanism of the Cr(III)/PAA system; (b) The Cr(III)/PAA system's TMP degradation process is illustrate.

Tables:

Table S1. Consensus mechanism and controversy summary of reactive oxygen in PAA activation system

Activation system	Consensus/main ROS	Major Controversies	Ignored ROS	Ref.
UV/PAA	HO·, $CH_3C(O)O$·	The relative contribution of $CH_3C(O)O$· and HO· to the degradation of pollutants changes with water quality conditions (pH, coexisting anions) and the structure of pollutants. Direct detection and quantitative difficulties of short-life $CH_3C(O)O$·.	$^1O_2$ (especially when pollutants are used as accelerators).	[1-3]
MW/PAA	$^1O_2$,R-O·	The specific contribution of the "non-thermal effect" of microwaves to the ROS generation path is still controversial.The source of $^1O_2$ is the direct decomposition of PAA or the secondary reaction of R-O. It is not completely clear.	·OH generated by local hotspots may be underestimated	[4]

Transition metal phase (Co^{2+}/PAA)	$CH_3C(O)O\cdot$, $CH_3C(O)OO\cdot$	The relative contribution of $CH_3C(O)O\cdot$ and $HO\cdot$ to the degradation of pollutants changes with water quality conditions (pH, coexisting anions) and the structure of pollutants. Direct detection and quantitative difficulties of short-life $CH_3C(O)O\cdot$	M^{4+}=O (M stands for transition metals such as iron, cobalt, manganese, copper, etc.)	[5-8]
Transition metal inhomogeneous phase (such as $CoMn_2O_4$, FeOCl)	$CH_3C(O)O\cdot$, $CH_3C(O)OO\cdot$	The contribution ratio of surface-mediated direct electron transfer process and free radical process. The role of catalyst surface properties (defects, oxygen voids) in non-free radical paths (such as $^1O_2$ generation).	Surface activated complex (direct oxidation). $^1O_2$ (especially on catalysts rich in oxygen-rich voids)	[9,10]
Carbon material/PAA (such as CNT, rGO)	$CH_3C(O)O\cdot$, Electronic transfer	Whether the oxygen-containing functional groups on the surface of carbon materials promote or inhibit the generation of ROS remains to be determined.	$^1O_2$ (related to the degree of material defect and graphitization). Surface persistent free radicals	[11,12]

Inorganic anions/PAA (such as HPO_4^{2-})	HO·,R-O·	The universality of phosphate radical activation of PAA and its effectiveness in different water quality contexts are controversial. The generated ROS type is highly dependent on pH and phosphate morphology, and the mechanism has not been unified.	Direct oxidation of phosphate radical-PAA complex	[13,14]

Table S2. Application challenges and countermeasures of activated peroxyacetic acid (PAA) technology in different environmental substrates

Stroma/Application field	Core challenges	Solution strategy	Ref.
Wastewater	NO competes with target pollutants HO· and R-O· to reduce degradation efficiency. Cl reacts with HO· to produce active chlorine species (such as Cl·, $Cl_2·^-$)	Priority is given to activation systems based on organic free radicals (such as $CH_3C(O)OO·$) or non-free radical paths (such as $^1O_2$, electron transfer)	[2,11,15-17]
Groundwater	Mass transfer restrictions and pH close to neutrality in the underground environment are inherent challenges.	Design a catalyst that is highly efficient and insensitive to HCO_3^- at a near-neutral pH	[18-20]
Soil	The ROS (especially hydrophilic HO·) produced by PAA molecules and their activation is difficult to diffuse and eventually come into contact with pollutants.	Consider the use of fisi-chemical technologies such as surfactants and solvent rinsing	[21-24]
Disinfection and sterilization	PAA and its ROS (such as HO·, R-O·) can oxidize Br-− in water into hybromoic acid, thus producing the carcinogen bromate;	Research and optimize the amount of PAA addition and contact time, and minimize the generation of brominate and organic by-products while ensuring the disinfection effect.	[25-27]

Table S3. Comparative analysis of PAA-induced transformation products and original ECs

Comparison project	Original ECs	Transformation products induced by PAA	Core risk differences and knowledge gaps	Ref.
Acute/chronic toxicity	It has the inherent toxicity of parent compounds (such as endocrine interference and antibiotic activity).	It may be higher or lower: a. Detoxification: mineralization into CO_2 and H_2O, or produces short-chain carboxylic acids with less toxicity. Poisoning: may produce intermediates (such as quinones and epoxy compounds) that are more toxic than the parent.	Most studies only focus on the maternal removal rate and lack a systematic evaluation of the toxicity of intermediate products.	[4,28]
Environmental durability	It usually has anti-biodegradation and chemical stability, and can exist in the environment for a long time.	Decrease: Open-ring and breaking bonds to produce smaller molecules that are more biodegradable. Maintain or increase: generate "tenacious" intermediates with a more stable structure and more difficult to further degrade.	There is insufficient research on the further convergence and degradation path of transformed products in the real environment (such as sewage treatment plants and rivers).	[21]
Potential for the formation	The parent compound itself	Significant risk: a. Active chlorine/bromine species production: PAA and its free radicals (such as	One of the most ignored key risks of PAA-AOPs. The existing research mostly	[2,15,16,29]

of halogenated organic matter (in the presence of Cl^-/Br^-)	may not contain halogen.	$CH_3C(O)O\cdot$) can oxidize Cl^-/Br^- to produce HOCl/HOBr and halogen free radicals. Halogen by-products: The reaction of the above-mentioned active halogen species with organic matter or intermediates may produce halogenated acetic acid, halogenated acetonitrile, bromate or even halogenated aromatic compounds.	focuses on disinfection by-products (such as trihalogen methane and haloacetic acid), while the identification, toxicity and generation mechanism of specific halogenation products generated by PAA-AOPs when treating complex wastewater are blank.

Table S4. Comparison of occupational exposure and storage risks of high-concentration PAA and H_2O_2/PMS/PDS

Risk category	High concentration PAA	H_2O_2 (concentrated solution, concentration >30%	PMS/PDS (solid or concentrated solution)	Core comparison and key risk points	Ref.
Major physical hazards	Explosive and unstable.Strong oxidation, volatility and flammability	Strong oxidizing agent, decomposition to produce oxygen, relatively stable	Strong oxidizing agent with good stability	PAA has the highest comprehensive risk. Its inherent instability and volatility constitute a unique risk, and the risk of H_2O_2 mainly comes from the decomposition of oxygen production, and PMS/PDS is mainly the conventional risk of solid oxidizing agents.	**[30,31]**
Health hazards (acute exposure)	Strong corrosive, high inhalation toxicity, suspected carcinogenic/mutagenic	Corrosive and irritating	Corrosive and irritating	PAA has the greatest potential for instant damage. Its vapor corrosion and inhalation toxicity are particularly prominent. In contrast, the harm of H_2O_2 and PMS/PDS is more due to contact corrosion.	**[25,26,32]**
Core requirements for storage	Temperature: Must be stored at low temperature (2-4° C) and away from heat sources.	Avoid light and store in a cool and ventilated place. Use a special	Sealed and moisture-proof, keep it in a dry and cool place. Store separately from reducing agents and	PAA has the most demanding storage conditions and the highest cost. It has the characteristics of forced low-	**[33,34]**

	Container: inert material, avoid using metal catalyst containers.	container with a pressure relief valve to avoid contact with metal impurities. Isolate from organic matter.	flammable materials.	temperature refrigeration and extreme sensitivity to pollution.

References:

1. Hollman, J.; Dominic, J.A.; Achari, G. Degradation of pharmaceutical mixtures in aqueous solutions using UV/peracetic acid process: Kinetics, degradation pathways and comparison with UV/H_2O_2. *Chemosphere* **2020**, *248*, 12
2. Ao, X.W.; Eloranta, J.; Huang, C.H.; Santoro, D.; Sun, W.J.; Lu, Z.D.; Li, C. Peracetic acid-based advanced oxidation processes for decontamination and disinfection of water: A review. *Water Res.* **2021**, *188*, 23
3. Cai, M.; Sun, P.; Zhang, L.; Huang, C.-H. UV/Peracetic Acid for Degradation of Pharmaceuticals and Reactive Species Evaluation. *Environmental Science & Technology* **2017**, *51*, 14217-14224
4. Dai, Y.H.; Qi, C.D.; Cao, H.; Wen, Y.N.; Zhao, Y.J.; Xu, C.M.; Yang, S.G.; He, H. Enhanced degradation of sulfamethoxazole by microwave-activated peracetic acid under alkaline condition: Influencing factors and mechanism. *Sep. Purif. Technol.* **2022**, *288*, 8
5. Rokhina, E.V.; Makarova, K.; Golovina, E.A.; Van As, H.; Virkutyte, J. Free Radical Reaction Pathway, Thermochemistry of Peracetic Acid Homolysis, and Its Application for Phenol Degradation: Spectroscopic Study and Quantum Chemistry Calculations. *Environmental Science & Technology* **2010**, *44*, 6815-6821
6. Kim, J.; Du, P.H.; Liu, W.; Luo, C.; Zhao, H.; Huang, C.H. Cobalt/Peracetic Acid: Advanced Oxidation of Aromatic Organic Compounds by Acetylperoxyl Radicals. *Environmental Science & Technology* **2020**, *54*, 5268-5278
7. Wang, Z.P.; Wang, J.W.; Xiong, B.; Bai, F.; Wang, S.L.; Wan, Y.; Zhang, L.; Xie, P.C.; Wiesner, M.R. Application of Cobalt/Peracetic Acid to Degrade Sulfamethoxazole at Neutral Condition: Efficiency and Mechanisms. *Environmental Science & Technology* **2020**, *54*, 464-475
8. Kim, J.; Zhang, T.Q.; Liu, W.; Du, P.H.; Dobson, J.T.; Huang, C.H. Advanced Oxidation Process with Peracetic Acid and Fe(II) for Contaminant Degradation. *Environmental Science & Technology* **2019**, *53*, 13312-13322
9. Zhang, L.L.; Chen, J.B.; Zheng, T.L.; Xu, Y.; Liu, T.C.; Yin, W.J.; Zhang, Y.L.; Zhou, X.F. Co-Mn spinel oxides trigger peracetic acid activation for ultrafast degradation of sulfonamide antibiotics: Unveiling critical role of Mn species in boosting Co activity. *Water Res.* **2023**, *229*, 12
10. Cheng, X.; Lian, J.; Jiang, M.; An, L.; Fan, Q.; Zeng, G.; Su, P.; Li, W.; Lv, Q.; Wu, Y.; et al. Unraveling the role of $CH_3C(=O)OO\cdot$ in the degradation of emerging organic contaminants via boosting activation of peracetic acid by iron oxychloride catalyst. *Sep. Purif. Technol.* **2024**, *330*, 125535
11. Kong, D.Z.; Zhao, Y.M.; Guo, H.D.; Han, M.; Fan, X.R.; Li, J.K.; He, X.; Ma, J. Unveiling the Direct Electron Transfer Regime of Peracetic Acid Activation: Quantitative Structure-Activity Relationship Analysis of Carbon Nanotube Catalysis. *ACS ES&T Eng.* **2023**, *3*, 1030-1041
12. Ren, H.T.; Qi, F.; Labidi, A.; Zhao, J.J.; Wang, H.; Xin, Y.; Luo, J.M.; Wang, C.Y. Chemically bonded carbon quantum dots/Bi2WO6 S-scheme heterojunction for boosted photocatalytic antibiotic degradation: Interfacial engineering and mechanism insight. *Applied Catalysis B-Environment and Energy* **2023**, *330*, 14
13. Deng, J.W.; Wang, H.B.; Fu, Y.S.; Liu, Y.Q. Phosphate-induced activation of peracetic acid for diclofenac degradation: Kinetics, influence factors and mechanism. *Chemosphere* **2022**, *287*, 9
14. Duan, P.J.; Liu, X.N.; Liu, B.H.; Akram, M.; Li, Y.W.; Pan, J.W.; Yue, Q.Y.; Gao, B.Y.; Xu, X.

Effect of phosphate on peroxymonosulfate activation: Accelerating generation of sulfate radical and underlying mechanism. *Appl. Catal. B-Environ. Energy* **2021**, *298*, 10
15. Chen, S.; Cai, M.Q.; Liu, Y.Z.; Zhang, L.Q.; Feng, L. Effects of water matrices on the degradation of naproxen by reactive radicals in the UV/peracetic acid process. *Water Res.* **2019**, *150*, 153-161
16. Wang, S.X.; Wang, H.B.; Liu, Y.Q.; Fu, Y.S. Effective degradation of sulfamethoxazole with Fe2+-zeolite/peracetic acid. *Sep. Purif. Technol.* **2020**, *233*, 8
17. Zhang, L.L.; Chen, J.B.; Zhang, Y.L.; Xu, Y.; Zheng, T.L.; Zhou, X.F. Highly efficient activation of peracetic acid by nano-CuO for carbamazepine degradation in wastewater: The significant role of H2O2 and evidence of acetylperoxy radical contribution. *Water Res.* **2022**, *216*, 10
18. Dai, C.; Li, S.; Duan, Y.; Leong, K.H.; Liu, S.; Zhang, Y.; Zhou, L.; Tu, Y. Mechanisms and product toxicity of activated carbon/peracetic acid for degradation of sulfamethoxazole: implications for groundwater remediation. *Water Res.* **2022**, *216*, 118347
19. Lin, J.B.; Hu, Y.Y.; Xiao, J.Y.; Huang, Y.X.; Wang, M.Y.; Yang, H.Y.; Zou, J.; Yuan, B.L.; Ma, J. Enhanced diclofenac elimination in Fe(II)/peracetic acid process by promoting Fe(III)/Fe(II) cycle with ABTS as electron shuttle. *Chem. Eng. J.* **2021**, *420*, 10
20. Zhao, M.; Yin, W.; Xiao, J.; Dong, J.; Deng, J.; Li, L.; Li, X.; Li, X.; Peng, B.; Dong, H. Peracetic acid activation by chitosan-derived nitrogen-doped carbon spheres loaded with zero-valent copper for efficient sulfamethazine degradation in groundwater. *Sep. Purif. Technol.* **2025**, *358*, 130291
21. N'Guessan, A.L.; Carignan, T.; Nyman, M.C. Optimization of the Peroxy Acid Treatment of α-Methylnaphthalene and Benzo[a]pyrene in Sandy and Silty-Clay Sediments. *Environmental Science & Technology* **2004**, *38*, 1554-1560
22. Levitt, J.S.; N'Guessan, A.L.; Rapp, K.L.; Nyman, M.C. Remediation of α-methylnaphthalene-contaminated sediments using peroxy acid. *Water Res.* **2003**, *37*, 3016-3022
23. N'Guessan, A.L.; Levitt, J.S.; Nyman, M.C. Remediation of benzo(a)pyrene in contaminated sediments using peroxy-acid. *Chemosphere* **2004**, *55*, 1413-1420
24. Scott Alderman, N.; N'Guessan, A.L.; Nyman, M.C. Effective treatment of PAH contaminated Superfund site soil with the peroxy-acid process. *J. Hazard. Mater.* **2007**, *146*, 652-660
25. Luukkonen, T.; Pehkonen, S.O. Peracids in water treatment: A critical review. *Crit. Rev. Environ. Sci. Technol.* **2017**, *47*, 1-39
26. Dunkin, N.; Weng, S.C.; Schwab, K.J.; McQuarrie, J.; Bell, K.; Jacangelo, J.G. Comparative Inactivation of Murine Norovirus and MS2 Bacteriophage by Peracetic Acid and Monochloramine in Municipal Secondary Wastewater Effluent. *Environmental Science & Technology* **2017**, *51*, 2972-2981
27. Luo, M.; Zhou, H.; Zhou, P.; Lai, L.; Liu, W.; Ao, Z.; Yao, G.; Zhang, H.; Lai, B. Insights into the role of in-situ and ex-situ hydrogen peroxide for enhanced ferrate(VI) towards oxidation of organic contaminants. *Water Res.* **2021**, *203*, 117548
28. Wang, J.W.; Wan, Y.; Ding, J.Q.; Wang, Z.P.; Ma, J.; Xie, P.C.; Wiesner, M.R. Thermal Activation of Peracetic Acid in Aquatic Solution: The Mechanism and Application to Degrade Sulfamethoxazole. *Environmental Science & Technology* **2020**, *54*, 14635-14645
29. Shah, A.D.; Liu, Z.Q.; Salhi, E.; Höfer, T.; von Gunten, U. Peracetic Acid Oxidation of Saline Waters in the Absence and Presence of H2O2: Secondary Oxidant and Disinfection Byproduct Formation. *Environmental Science & Technology* **2015**, *49*, 1698-1705

30. Saha, M.S.; Denggerile, A.; Nishiki, Y.; Furuta, T.; Ohsaka, T. Synthesis of peroxyacetic acid using in situ electrogenerated hydrogen peroxide on gas diffusion electrode. *Electrochem. Commun.* **2003**, *5*, 445-448
31. Llanos, J.; Moraleda, I.; Sáez, C.; Rodrigo, M.A.; Cañizares, P. Optimization of a cell for the electrochemical synergistic production of peroxoacetic acid. *Electrochim. Acta* **2018**, *260*, 177-183
32. Koivunen, J.; Heinonen-Tanski, H. Inactivation of enteric microorganisms with chemical disinfectants, UV irradiation and combined chemical/UV treatments. *Water Res.* **2005**, *39*, 1519-1526
33. Cai, M.Q.; Sun, P.Z.; Zhang, L.Q.; Huang, C.H. UV/Peracetic Acid for Degradation of Pharmaceuticals and Reactive Species Evaluation. *Environmental Science & Technology* **2017**, *51*, 14217-14224
34. Miklos, D.B.; Remy, C.; Jekel, M.; Linden, K.G.; Drewes, J.E.; Hübner, U. Evaluation of advanced oxidation processes for water and wastewater treatment – A critical review. *Water Res.* **2018**, *139*, 118-131
